# A probabilistic hazard and risk assessment of exposure to metals and organohalogens associated with a traditional diet in the Indigenous communities of *Eeyou Istchee* (northern Quebec, Canada)

**DOI:** 10.1007/s11356-022-23117-2

**Published:** 2022-09-24

**Authors:** Robert J. Moriarity, Leonard J. S. Tsuji, Eric N. Liberda

**Affiliations:** 1 School of Occupational and Public Health, Toronto Metropolitan University, Toronto, ON Canada; 2 Department of Health and Society, University of Toronto, Toronto, ON Canada; 3 Department of Physical and Environmental Sciences, University of Toronto, Toronto, ON Canada

**Keywords:** Metals, Organohalogens, Toxicological risk assessment, Traditional food, Cree Peoples, James Bay, Canada

## Abstract

Hunting, trapping, and fishing are part of an Indigenous lifestyle in subarctic Canada. However, this lifestyle may be a route of exposure to contaminants and may pose a risk for the people who rely on a subsistence diet. Monte Carlo simulations for the chemical concentration of eight game species and one fish species were carried out by randomly sampling 10,000 samples from the contaminant measures for each species. We then calculated a probabilistic non-carcinogenic hazard quotient or carcinogenic risk values to estimate the human health risk of exposure to contaminants. Of the species examined, ducks were of concern for potential carcinogenic risk. There was a modeled probabilistic 95^th^-percentile risk associated with the consumption of ducks and polybrominated biphenyl (PBB) congener 153 in boys, women, and men (1.09 × 10^−6^, 1.57 × 10^−6^, and 2.17 × 10^−6^, respectively) and, to a lesser extent, with geese and polychlorinated biphenyl (PCB) congener 153 in men (1.19 × 10^−6^). Contaminant concentration in the intake rate (food consumption) for PBB congener 153 exposure risk was more important than that for PCB congener 153, where intake rate had greater relevance. The consumption of waterfowl may increase the exposure to organohalogens; however, there are health and wellness benefits associated with the harvesting and consumption of subsistence foods that must also be considered. We recommend follow-up species-specific studies focused on ducks to clarify and elucidate the results in the present study.

**Supplementary Information:**

The online version contains supplementary material available at 10.1007/s11356-022-23117-2.

## Introduction

Hunting, trapping, and fishing are central to the Cree Peoples’ way of life, and these activities provide a variety of traditional foods (Tsuji et al. 2020). Moreover, the consumption of traditional foods as part of a subsistence lifestyle affords nutrients that are limited in the western diet (e.g., omega-3 and omega-6 polyunsaturated fatty acids (Ebbesson et al. 2005; Valera et al. 2011a)). Noreen et al. (2018) found that traditional foods were consumed frequently in our study region, up to three times per week; however, up to 39% of the diet also came from processed western foods—this is comparable to other Indigenous communities in Canada (Batal et al. 2018; Ramirez Prieto et al. 2022). Diets rich in traditional foods help to offset the consumption of western foods low in nutritional value that may lead to adverse health impacts (i.e., type 2 diabetes, cardiovascular and neurological diseases (Gates et al. 2016; Jeppesen et al. 2014; Munch-Andersen et al. 2012; Popeski et al. 1991)). Additionally, the Cree Peoples’ subsistence lifestyle helps offset the high cost of imported foods to the Canadian north (Gates et al. 2012). Nonetheless, traditional food consumption is also a potential route of exposure to metals and organic contaminants from a legacy of chemical use, industry, and resource development in Indigenous territories, and these foods can act as potential sources of contaminant exposure (Belinsky and Kuhnlein 2000; Horak et al. 2014; Liberda et al. 2019; Liberda et al. 2018; Moriarity et al. 2020a; Nieboer et al. 2017; Ratelle et al. 2020; Valera et al. 2011b, 2013). For example, in our study region, traditional foods such as fish (e.g., walleye, lake trout) have been found to contain high levels of mercury (Hg) (Moriarity et al. 2020a), game (e.g., hare) contains high levels of lead (Pb), and birds (e.g., ptarmigan and duck) contain high levels of organohalogens (Chan et al. 2021). These contaminants have been found in the blood of community members living in our study region, therefore suggesting some potential exposure from traditional food consumption (Liberda et al. 2021a). Atmospheric long-range transport of contaminants is also an issue many Indigenous communities face in Canada (Pelletier et al. 2021; Wong et al. 2018)—and globally (Kyllönen et al. 2020)—and is thought to contribute to the environmental contamination of traditional territories.

Environmental contaminants impact subarctic and arctic communities at disproportionately higher rates than the urban population in Canada because exposure to contaminants is primarily, but not entirely, from the consumption of traditional foods that have bioaccumulated and biomagnified contaminants up the food chain (Liberda et al. 2018; Moriarity et al. 2020b; Moriarity et al. 2020a; Tsuji et al. 2007a). Some of these contaminants have been known to be neurotoxic (e.g., Hg, Pb) (Boucher et al. 2009; Ijomone et al. 2020), endocrine disruptors (e.g., polychlorinated biphenyls (PCBs)) (Bimonte et al. 2021; Khare et al. 2020; Wainman et al. 2016; Yilmaz et al. 2020), and potentially carcinogenic (e.g., arsenic (As)) (Koual et al. 2019; Park et al. 2020). Essential metals (e.g., copper (Cu), selenium (Se)) are important for good health if consumed within tolerable limits, but non-essential metals (e.g., As, cadmium (Cd)) may be toxic and may have a negative impact on health if consumed in quantities that exceed safety thresholds (e.g., neurotoxicity, cognitive impairment, carcinogenicity) (Tchounwou et al. 2012). Additionally, recent research further indicates that metal exposure not exceeding national or international guidelines may also illicit negative affect health effects (e.g., hepatic, vascular) (Urbano et al. 2022; Urbano et al. 2021). Likewise, organohalogens (e.g., PCBs) and pesticides (e.g., dichlorodiphenyltrichloroethane (DDT)) are of concern because they persist in the environment. Since these compounds have high lipophilicity, they can be stored in the fat of traditional foods and, ultimately, in humans following consumption and are associated with reproductive and carcinogenic effects (Adamou et al. 2020; Agus et al. 2021; Kartalović et al. 2020; Khare et al. 2020; Reyes et al. 2015; Wainman et al. 2016). Therefore, there may be a cause for concern for the potential health hazard or risk of exposure to these contaminants from the consumption of traditional foods.

In light of these concerns—and a paucity of studies employing unique modeling of probable exposure to contaminants from traditional food consumption in Indigenous communities—the purpose of this study was to examine the potential routes of exposure to metals and organohalogens from the consumption of traditional foods in the Cree traditional territory of *Eeyou Istchee* in northern Quebec, Canada, and evaluate the hazard quotient or carcinogenic risk to *Eeyouch* (Cree Peoples) of this territory who consume traditional foods as part of a subsistence lifestyle. The ultimate goal was to provide hazard or risk information to *Eeyouch* in this territory who consume traditional foods and enable community members to make informed decisions for their health.

## Methods

### Study region

**Fig. 1:**
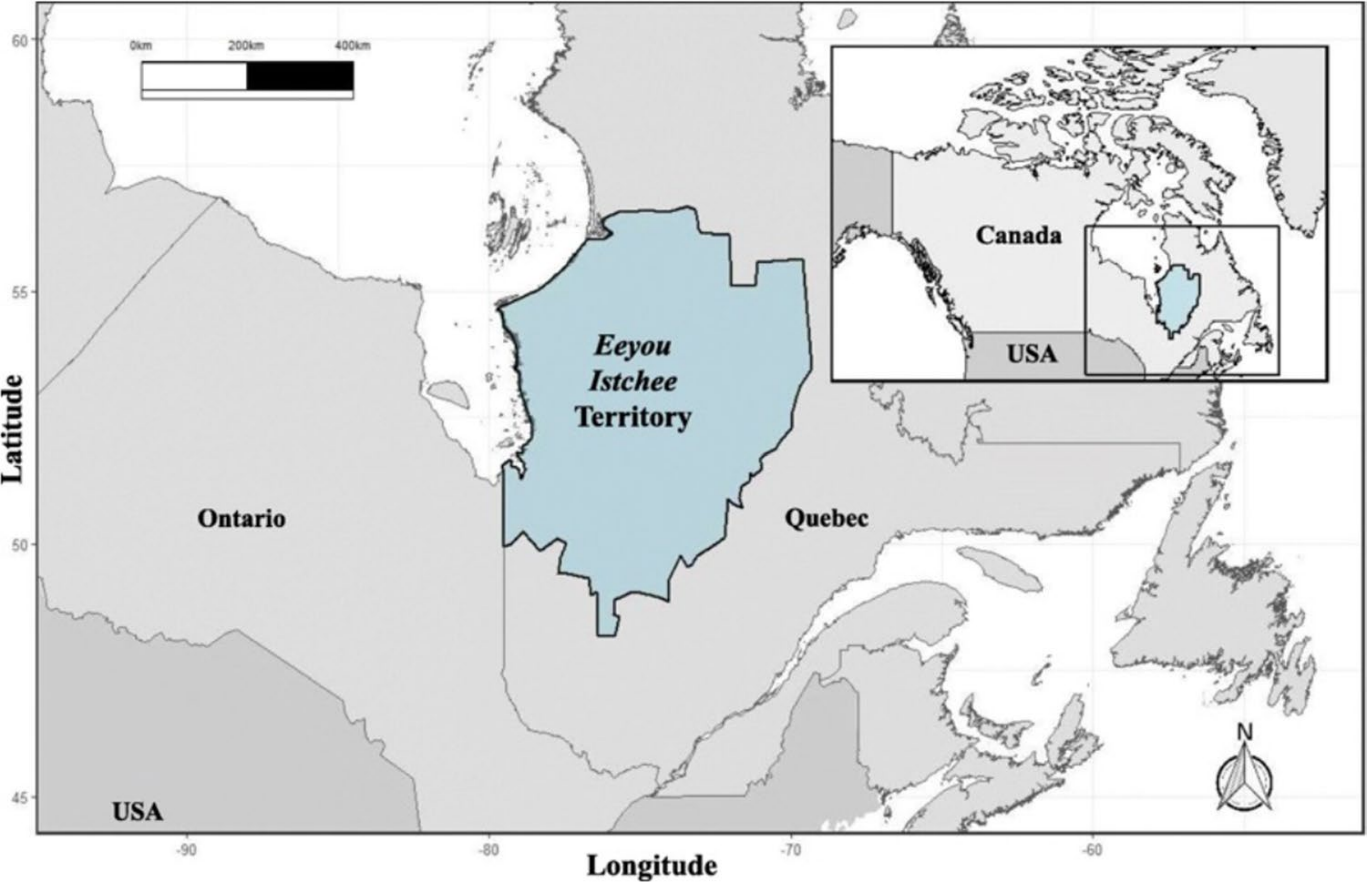
The *Eeyou Istchee* territory of northern Quebec, Canada. Adapted from Liberda et al. (2019)

The nine Cree communities of eastern James and Hudson Bay, Quebec, make up the traditional territory known as the Eeyou Istchee (currently, eleven communities; Fig. 1). These communities vary in isolation, with inland communities accessible by road, air, and water, and some coastal communities only accessible by air and water. Traditional activities vary by community, but hunting remains an essential aspect of traditional practices in this region (Gaudin et al. 2014; Giroux et al. 2022). For this study, under the guidance of the Cree Trappers’ Association, we collected meat samples from traditional wild game harvested by the Cree of Whapmagoostui, Chisasibi, Wemindji, Eastmain, Waskaganish, Nemaska, Waswanipi, Ouje-Bougoumou, and Mistissini First Nations.

### Sample collection

Aseptic sampling kits were assembled and shipped to each Cree Trappers’ Association representative in each community. These kits consisted of wide-mouth Nalgene™ sample bottles, nylon gloves, sample bags, scalpel blades, pre-made labels, sample information sheets, and indelible markers. Training for aseptic field techniques was provided to the Cree Trappers’ Association representatives, and additional instructions were provided to all hunters who provided samples for analysis.

### Sample analysis

#### Metal contaminant analysis

All tissue samples were analyzed at the *Centre de Toxicologie du Québec* (CTQ), *Institut National de Santé Publique du Québec* (INSPQ), an ISO 17025–accredited laboratory. Tissue samples were initially lyophilized with 10.00 grams (g) placed in a Nalgene™ flask and then frozen at − 80 °C, followed by freeze-drying for 48 h. The tissue was ground prior to weighing for digestion. Digestion of lyophilized tissues (200 milligrams (mg)) was performed using concentrated nitric acid (2.00 milliliters (mL), ultrapure). The digest was diluted with 200 microliters (μL) of concentrated hydrochloric acid and 5.00 mL of diluent (aqueous solution containing 0.002% (m/v) l-(+)-cysteine and 100 micrograms (μg)/L (v/v) gold, and 100 μL ethanol). Germanium, indium, platinum, rhenium, rhodium, and platinum-terbium (1.00 mL of 0.20 μg/mL) were added as internal standard. Prior to ICP-MS analysis, 2 mL of the diluted digest was further diluted with 7.90 mL of diluent and 100 μL of ethanol.

Twenty-four metals (aluminum (Al), antimony (Sb), As, barium (Ba), beryllium (Be), bismuth (Bi), Cd, cesium (Cs), chromium (Cr), cobalt (Co), Cu, Pb, manganese (Mn), Hg, molybdenum (Mo), nickel (Ni), Se, silver (Ag), thorium (Th), tin (Sn), uranium (U), vanadium (V), tellurium (Te), and thallium (Tl)) were analyzed on a single-quadrupole ICP-MS (Elan DRC II from Perkin Elmer) with autosampler ESI-SC-4 and workstation Elan version 3.0.

The method was developed and validated for monitoring purposes following ISO 17025 guidelines. Limits of detection were determined using 3 standard deviations from 10 consecutive measurements of a representative sample and varied from 6.40 × 10^−4^ mg/kg (Cs) to 0.50 mg/kg (Al). Quality control was performed using certified reference material (DOLT-5 from NRC and NIST 1640a from NIST) and laboratory-made reference material (FANI 1207) after every 10th sample and at the end of each analytical sequence.

#### Organic contaminant analysis

As with metal contaminants, organic analysis was also performed at the INSPQ. All tissue samples were assessed for PCB congeners (28, 52, 99, 101, 105, 118, 128, 138, 153, 156, 163, 170, 180, 183, 187, 194, and 209), β-HCH, α-chlordane, γ-chlordane, oxychlordane, *cis*-nonachlor, *trans*-nonachlor, mirex, *o*,*p*′-DDE, *p*,*p*′-DDE, *o*,*p*′-DDT, *p*,*p*′-DDT, *o*,*p*′-DDD, *p*,*p*′-DDD, Parlar 26, Parlar 32, Parlar 50, Parlar 62, polybrominated biphenyl (PBB) 153, polybrominated diphenyl ether (PBDE) 47, PBDE 99, PBDE 100, PBDE 153, and PBDE 154. Sample weight varied by the nature of the matrix and fat content and ranged from 300 to 500 mg of fish and animal tissues. All tissue samples were lyophilized and mixed with 200 μL of hexane followed by enrichment with internal standards (α-HCH-13C6, β-HCH-13C6, oxychlordane-13C10, *trans*-nonachlor-13C10, *o*,*p*′-DDE-13C12, *o*,*p*′-DDD-13C12, *o*,*p*′-DDT-13C12, *p*,*p*′-DDE-13C12, *p*,*p*′-DDD-13C12, PCB 28-13C12, PCB 52-13C12, PCB 118-13C12, PCB 141-13C12, PCB 153-13C12, PCB 180-13C12, PCB 194-13C12, Parlar 26-13C10, Parlar 50-13C10, PBDE 77-13C12, and PBDE 153-13C12) prior to the addition of 10 mL of acetonitrile. Vortexed samples were extracted using ultrasonication before chemically drying using a magnesium sulfate and sodium acetate mixture. The samples were then extracted and purified by the QuEChERS methodology with the Supel QuE Z-Sep+ extraction powder (Supelco/Sigma-Aldrich, Oakville, Ontario, Canada), then evaporated to dryness. After reconstitution in 1.00 mL of hexane, the samples were purified on silica gel cartridges and eluted with 5.00 mL of dichloromethane:hexane (25:75) mixture. Before injection in the APGC-MS/MS, samples were evaporated to dryness and reconstituted in 300 μL of hexane.

Sample extract (1.00 μL) was assessed on an APGC-MS/MS with an Agilent 7890B gas chromatograph (GC; Agilent Technologies, Mississauga, Ontario, Canada) coupled with a Waters Xevo TQ-XS tandem mass spectrometer (MS/MS; Waters, Milford, MA, USA) and fitted to the MS/MS with an Agilent 30m DB-XLB column (0.25 millimeters (mm) i.d., 0.10 μm film thickness) with a run time of 41.93 min. Ions were measured in multiple reaction monitoring post-atmospheric pressure ionization in positive mode. Lipid content was determined using gravimetry with 500 mg of a tissue sample. The limits of detection (LODs) for all compounds ranged from 3.30 × 10^−2^ to 0.27 μg/kilogram (kg). The LOD was determined using a signal-to-noise ratio of 3, and quality control was assessed using fish tissue (SRM-1947) containing almost all the analyzed compounds and was provided by the National Institute of Standards & Technology (NIST; Gaithersburg, MD, USA).

### Statistical analysis

Data analysis was initially limited to traditional foods with samples of *n* ≥ 5; this criterion yielded eight traditional wild game meats and one species of fish for a total of 151 samples (*n* = 151) (Table 1). The traditional foods included bear (*Ursus americanus*), beaver (*Castor canadensis*), caribou (*Rangifer tarandus*), duck (*Anatidae* spp.), goose (*Anser* spp.), moose (*Alces alces*), grouse (*Tetraonini* spp.), hare (*Lepus americanus*), and walleye (*Sander vitreus*). Data values for each contaminant less than the minimum level of detection (MLOD) were assigned numerical values of 50% of the MLOD (US Environmental Protection Agency (EPA) 2000; US EPA 2015a) to allow for statistical analyses. R version 3.6.3 (R Core Team 2020) was used to determine the arithmetic mean and standard deviation, minimum, 5^th^ to 99^th^ percentiles, and maximum value for each contaminant.

**Table 1:** Contaminants included in the probabilistic hazard or risk examinations by traditional food

Traditional food	Contaminant	
	Metals	Organohalogens
Bear (*n* = 15)	Al, Ba, Cd, Cr, Cu, Pb, Hg, Mo, Ni, Se	PCB 153, PCB 170, PCB 180, PCB 52
Beaver (*n* = 22)	Al, Cd, Cr, Cu, Pb, Hg	–
Caribou (*n* = 5)	Ba, Cd, Cu, Pb, Hg, Se	–
Duck (*n* = 7)	Al, Ba, Cd, Cr, Cu, Pb, Hg, Mo, Se	*Cis*-nonachlor, mirex, oxychlordane, PBB 153, PBDE 100, PBDE 153, PBDE 154, PBDE 47, PCB 101, PCB 118, PCB 128, PCB 138, PCB 153, PCB 156, PCB 163, PCB 170, PCB 180, PCB 183, PCB 187, PCB 194, PCB 28, PCB 52, PCB 99, *p*,*p*′-DDD, *p*,*p*′-DDE, *p*,*p*′-DDT, β-hexachlorocyclohexane, toxaphene Parlar 26, toxaphene Parlar 50, *trans*-nonachlor
Goose (*n* = 23)	Sb, Ba, Cu, Pb, Mo, Se	PCB 153, *p*,*p*′-DDE
Grouse (*n* = 13)	Ba, Cd, Cu, Pb, Mo, Se, Sn	–
Hare (*n* = 19)	Ba, Cd, Cr, Cu, Se	–
Moose (*n* = 37)	Al, Ba, Cd, Cr, Cu, Pb, Se	–
Walleye (*n* = 10)	Ba, Cu, Pb, Hg, Se, Sn	*Cis*-nonachlor, PBDE 100, PBDE 47, PBDE 99, PCB 118, PCB 128, PCB 138, PCB 153, PCB 170, PCB 180, PCB 187, *p*,*p*′-DDE

Metals: *Al* aluminum, * Sb* antimony, *Ba* barium, *Cd* cadmium, *Cr* chromium, *Cu* copper, *Pb* lead, *Hg* mercury, *Mo* molybdenum, *Ni* nickel, *Se* selenium, *Sn* tin; organohalogens: *PCB* polychlorinated biphenyl, *PBB* polybrominated biphenyl, *PBDE* polybrominated diphenyl ethers, *DDD* dichlorodiphenyldichloroethane, *DDE* dichlorodiphenyldichloroethylene, *DDT* dichlorodiphenyltrichloroethane

Traditional food contaminant data for both metals and organohalogens were further limited and subject to a probabilistic risk assessment calculation if the traditional food sample contained 50% or more valid detects (i.e., excluding non-detects and/or adjusted MLOD values), and the mean tissue concentration was at least 0.01 mg/kg for metals and 1.00 × 10^−4^ mg/kg for organohalogens. Contaminants meeting the criteria for each traditional food are summarized in Table 1.

### Probabilistic risk assessment

The consumption of the traditional foods was then used to determine the potential route of exposure for each contaminant. Consumption data of each traditional food by demographic was based on Health Canada (2007), suggesting a serving size of 75 g per serving, where girls and boys were assumed to eat one serving, women two servings (150 g), and men three servings (225 g). Additionally, the daily portions of these animal species were adapted from our previous work (Nieboer et al. 2013) from the monthly consumption of traditional foods in our study region. The consumption of these traditional foods in grams per day (g/day) by demographic is listed in Table 2.

**Table 2:** Consumption of traditional foods by demographic in the *Eeyou Istchee*

Traditional food	Mass of food consumed per day (g/day)			
	Girls	Boys	Women	Men
Bear	1.050	0.550	0.900	3.450
Beaver	1.280	0.950	3.800	8.850
Caribou	1.350	1.680	2.400	7.350
Duck	0.830	2.250	5.750	9.150
Goose	5.300	7.800	13.50	21.90
Grouse	1.100	1.430	3.100	8.400
Hare	1.330	2.280	5.150	11.70
Moose	3.130	3.800	12.80	19.65
Walleye	0.780	3.630	7.300	11.48

Deterministic hazard and risk assessments provide results that can be readily compared to a threshold to assess or evaluate contaminants’ potential harm or probability of risk of injury or cancer. However, deterministic approaches to hazard or risk assessment are limited as they only provide a static snapshot of the contaminant under investigation and can be easily skewed by extreme contaminant values and/or small sample sizes; therefore, a level of uncertainty arises in interpreting the results (Kirchsteiger 1999). Consequently, one way to reduce these uncertainties is to use Monte Carlo simulations to estimate the probabilistic hazard or risk in place of a deterministically derived approach. This resampling technique samples multiple iterations of the probability distribution of the contaminant tissue concentration data for each traditional food to acquire a range of probable outcomes that are then used to calculate hazard or risk (US EPA 2015b).

We used descdist in the fitdistrplus package (Delignette-Muller and Dutang 2015) in R version 3.6.3 to estimate the distribution of the chemical concentration in the traditional foods prior to running probabilistic simulations. Monte Carlo probabilistic simulations for the chemical concentration in the species were then carried out by randomly sampling 10,000 iterations from log-normal distributions resulting from the actual contaminant concentrations for each traditional food and then inputting this estimate into a calculation (Eq. 1) for chemical intake from food (The Agency for Toxic Substances and Disease Registry (ATSDR) 2019):

1 $$I\;\left(\frac{mg}{kg\;d}\right)=\frac{IR\;\left({\displaystyle\frac gd}\right)\times C\;\left({\displaystyle\frac{mg}{kg}}\right)\times EF\;\left({\displaystyle\frac dy}\right)\times ED(y)}{BW\;(kg)\times AT\;(d)}\times10^{-3}\left(\frac{kg}g\right)$$

where *I* is the intake of the chemical in milligrams per kilogram of body weight per day (mg/kg/d), *C* is the probabilistic chemical concentration in the animal species in milligrams per kilogram (mg/kg), IR is the intake rate of food in grams per day (g/d), EF is the exposure frequency in days per year (d/y), ED is the exposure duration in years (y), AT is the averaging time in days, and BW is the bodyweight in kilograms (kg). The exposure duration for girls and boys was the median age of the population at 9 years old and 40 years old for women and men as this is a relatively young population overall. Body weights in the calculation were normalized based using the following data from Moriarity et al. (2020b): 62.98 ± 21.41 kg for girls, 65.98 ± 23.14 kg for boys, 91.59 ± 20.37 kg for women, and 96.08 ± 18.56 kg for men as these measures are representative of consumers in our study region.

We then used the EnviroPRA (Barrio-Parra and Dominguez-Castillo 2017) package in R version 3.6.3 using the probabilistic chemical intake (*I*) for either a non-carcinogenic hazard quotient (HQ; Eq. 2) or risk (*R*; Eq. 3) depending on the type of contaminant to estimate a probability of the human health risk of exposure to contaminants from the intake of the nine traditional foods:

2 $$\textrm{HQ}=\frac{I\ }{\textrm{RfD}}$$

3 $$R=I\times \textrm{SF}$$

where RfD is the reference dose in mg/kg/day, and SF is the slope factor in (mg/kg/day)^−1^. The reference doses and slope factors for each contaminant are presented in Tables S1 and S2. We then determined the 5^th^ to 99^th^ percentiles for all HQ or *R* values; the set target for each HQ or *R* was 95^th^ percentile greater than 1.00 or 1.00 × 10^−6^, respectively, based on EPA (2005) guidelines for hazard and risk assessment. The ggplot2 (Wickham 2016) package in R was used to create boxplots for traditional foods where the 95^th^-percentile probabilistic risk exceeded 1.00 × 10^−6^. Additionally, a sensitivity analysis (Jiménez-Oyola et al. 2020; Öberg and Bergbäck 2005) was applied when the 95^th^-percentile HQ (HQ_95_) was greater than 1.00, or the 95^th^-percentile risk (*R*_95_) was greater than the lifetime attributable risk of 1.00 × 10^−6^ for each traditional food and contaminant. The sensitivity analyses were carried out using the Spearman’s rank correlation coefficient (*ρ*) using 10,000 iterations and *p* < 0.05.

## Results

### Contamination of traditional foods

The arithmetic means, standard deviation, minimum, 5^th^ to 99^th^ percentiles, and maximum value for each metal and organic contaminant are presented in Tables S3 and S4 for each of the nine traditional foods, respectively.

#### Metals

In all traditional foods, the descending order of metal concentrations was ranked as follows: Al > Cu > Se > Hg > Pb > Cd > Cr > Ba > Sn > Ni > Sb ≈ Mo. The highest median metal concentration was for Cu in goose (4.60 mg/kg), and the maximum metal concentration was for Al in moose (510.00 mg/kg). The median Se concentration was 1.60 mg/kg in duck, and the median Hg concentration was 0.58 mg/kg in walleye. The median concentrations of the remaining metals were all less than 0.22 mg/kg (beaver, Pb). The highest mean metal concentrations of Al and Cu were found in moose at 29.09 mg/kg and 12.16 mg/kg, respectively (Table S3). These metal concentrations were also elevated in bear and duck compared to other traditional foods’ metal mean concentrations. The mean metal concentration of Pb and Cd was highest in beaver at 0.88 mg/kg and 0.26 mg/kg, respectively; Ba was highest in hare (0.51 mg/kg); Sn was highest in grouse and walleye, equally (0.04 mg/kg); Ni was highest in and limited to tissue samples in bear (0.03 mg/kg); Sb was highest in and limited to tissue samples in goose (0.02 mg/kg); and Mo was equivalently highest in bear, duck, and goose (0.02 mg/kg).

#### Organohalogens

The highest mean and median concentrations of an organohalogen were for PCB congener 153 (mean: 0.56 mg/kg; median: 0.53 mg/kg) and *p*,*p*′-DDE (mean: 0.56 mg/kg; median: 0.54 mg/kg), respectively, in ducks. The highest concentration of an organohalogen was tied for PCB congener 153 and *p*,*p*′-DDE in ducks at 1.30 mg/kg. Overall, ducks had the highest concentrations of organohalogens compared to bears, geese, or walleye (Table S4). The other organohalogen mean and median concentrations were lower than approximately 0.38 mg/kg.

### Probabilistic risk assessment

#### Metals

In all traditional food samples, the resulting probabilistic 95^th^-percentile HQ (HQ_95_) for each modeled metal contaminant concentration was below 1.00 (Table S5).

#### Organohalogens

**Fig. 2:**
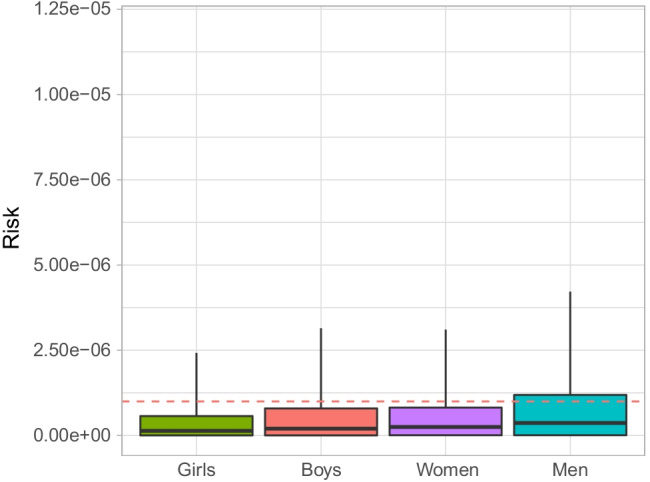
Carcinogenic probabilistic risk from the consumption of goose contaminated with PCB congener 153. The box on the boxplot has a lower probabilistic risk range of *P*_5_, a mid-range (black line) of *P*_50_, and an upper range of *P*_95_. The lower whiskers indicate minimum probabilistic risk, while the upper whiskers indicate maximum probabilistic risk. The dashed red line indicates a carcinogenic risk threshold of 1.00 × 10^−6^

**Fig. 3:**
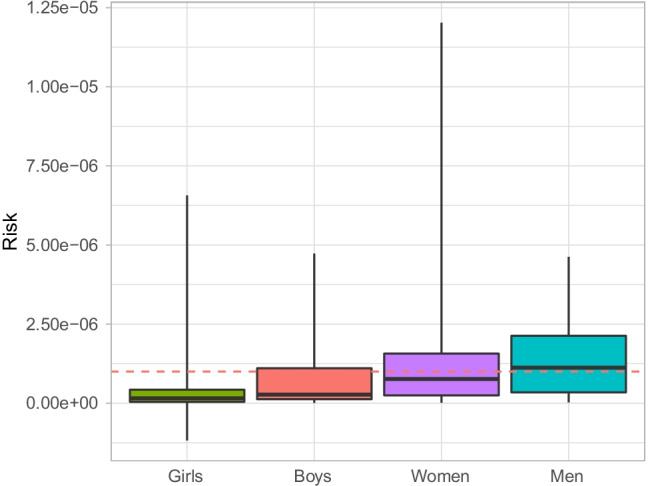
Carcinogenic probabilistic risk from the consumption of duck contaminated with PBB congener 153. The box on the boxplot has a lower probabilistic risk range of *P*_5_, a mid-range (black line) of *P*_50_, and an upper range of *P*_95_. The lower whiskers indicate minimum probabilistic risk, while the upper whiskers indicate maximum probabilistic risk. The dashed red line indicates a carcinogenic risk threshold of 1.00 × 10^−6^

**Fig. 4:**
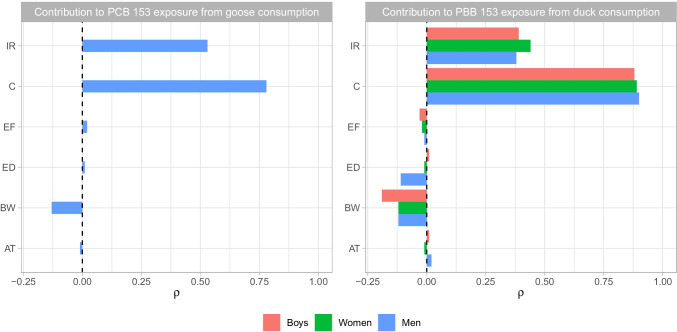
Sensitivity analysis results (Spearman’s *ρ*) for contribution to organohalogen exposure from traditional foods by a consumer group when the *R*_95_ was greater than 1.00 × 10^−6^

Two traditional foods, goose and duck, yielded a potential risk (*R*_95_ > 1.00 × 10^−6^) of exposure to organohalogens PCB congener 153 (Fig. 2) and PBB congener 153 (Fig. 3) for selected demographics. The remaining *R*_95_ for other organohalogens and traditional foods was less than 1.00 × 10^−6^ (Table S6). The *R*_95_ for the men’s consumption of goose was 1.19 × 10^−6^ (Fig. 2), and the sensitivity analysis (Fig. 4; Table S7) revealed that the parameters influencing a risk were the *C* of PCB congener 153 (*ρ* = 0.78) and the IR (*ρ* = 0.53); BW had less influence on risk (*ρ* = − 0.13). The *R*_95_ for the consumption of duck of boys, women, and men was 1.09 × 10^−6^, 1.57 × 10^−6^, and 1.19 × 10^−6^, respectively (Fig. 3). The parameters *C* and IR for PBB congener 153 had a positive influence on risk for boys, women, and men (*ρ* range *C*: 0.88–0.90; *ρ* range IR: 0.38–0.44), where BW had a less influence on risk for boys (*ρ* = − 0.19), and women and men (*ρ* = − 0.12) (Fig. 4; Table S7).

## Discussion

The modeled probabilistic exposure to contaminants from consuming traditional foods has been examined in the literature for the general population (Fakhri et al. 2018; Sanaei et al. 2021; Wong et al. 2020) and, to a lesser extent, Indigenous communities in Canada (Juric et al. 2018; Juric et al. 2017). Our study’s main results establish there is an increased risk of exposure to organohalogens for some of the demographics in our study region: men have a risk of exposure to PCB congener 153 from the consumption of goose, and boys, women, and men have a risk of exposure to PBB congener 153 from the consumption of duck. Overall, however, the hazard or risk of exposure to metals and other organohalogens from the traditional foods in the study region is encouragingly low.

This study is beneficial in that it adds new information that aids to satisfy the knowledge gap of exposure to contaminants from consuming traditional foods in Indigenous communities and adds to the existing literature for modeled probabilistic exposure assessment. To our knowledge, this is one of the largest and most detailed contaminant and exposure studies in the study region. The outcomes of this study are crucial in communities where subsistence hunting, trapping, and fishing are carried out. However, we note here that the estimated portion sizes based on Health Canada (2007) may have influenced the intake and, thus, potential exposure to contaminants in our study. Therefore, we urge a precautionary approach to interpreting the results of this preliminary study.

### Characterizing risk from metals

Exposure to metals from traditional cultural foods in Indigenous communities has been found to be of relatively low risk in previous studies in our study region (Belinsky and Kuhnlein 2000; Liberda et al. 2021b; Nieboer et al. 2017). Similar results have been demonstrated in other Indigenous communities in Canada (Bordeleau et al. 2016) and in other parts of the world (Carpenter 2014). However, we note that low risk does not imply no risk. For example, the HQ_95_ for exposure to Hg from the consumption of walleye is approaching 1.00 for men, and though this is a known contaminant issue in the study region (Moriarity et al. 2020b; Moriarity et al. 2020a), it is important to highlight that exposure to Hg continues to be an issue in the study region and requires follow-up monitoring. Additionally, exposure to metals in Indigenous communities is linked to lifestyle factors (e.g., Cd) such as smoking (Charania et al. 2014; Ratelle et al. 2018) or the use of Pb ammunition (Liberda et al. 2018; Tsuji et al. 2008a, 2008b; Tsuji et al. 1999; Tsuji et al. 2009; Tsuji and Nieboer 1997) to hunt traditional foods and are therefore not directly related to a risk of exposure directly from a contaminated natural environment. Additionally, since the consumption of these traditional foods does not demonstrate metal exposure hazards in our present study, the discussion of our results is therefore focused on the risk of exposure to organohalogens.

### Characterizing risk from organohalogens

Assessing risk following exposure to organohalogens is complex as exposure to these contaminants is not in isolation but to a mixture of contaminants rather than a single contaminant at one time (Akbar et al. 2021; Aminov et al. 2016; Aminov and Carpenter 2020; Liberda et al. 2021a). However, since many organohalogens have similar health effects and some have established dietary routes of exposure (that is fish and exposure via airborne pathways) (Weitekamp et al. 2022), we can characterize the risk of traditional food consumption in our study region. This approach has been used in previous studies (Huang et al. 2022; Naqvi et al. 2020; Sari and Esen 2022). Therefore, investigating any route of exposure from organohalogens is critical to estimating the potential risk to humans following exposure from consuming contaminated food.

A risk analysis is more specific than a hazard analysis because the risk is a quantifiable probability and hazard is not (Lofstedt 2011). Our study’s results demonstrate a slightly increased risk of exposure to PCB congener 153 for the male consumption of goose (Fig. 2). There is a slightly increased risk of exposure to PBB congener 153 for boys’ consumption of ducks and an increased risk of exposure to PBB congener 153 (Fig. 3) from the consumption of ducks for women and men. Previous studies have indicated the presence of these organohalogens in geese and ducks (Ali et al. 2017; Horak et al. 2014; Pesiakova et al. 2018; Tomza-Marciniak et al. 2019); however, there are no studies that we are aware of assessing the risk of consuming contaminated geese and ducks for Indigenous community members who rely on traditional foods as part of a traditional cultural diet, as in Eeyou Istchee. Furthermore, the overall risk following exposure to other organohalogens from the traditional foods in our study region is below the lifetime attributable risk of 1.00 × 10^−6^; therefore, we limit our discussion of risk of exposure from traditional food consumption to an *R*_95_ value greater than this threshold.

#### PCB congener 153 exposure and risk from goose consumption

PCBs are persistent organic pollutants that were banned from manufacture in the late 1970s in Canada and the United States (Canada 2009a; US EPA 2015c), but do not break down in the environment and, thus, persist and bioaccumulate in organisms (Erickson 2018; Khare et al. 2020). PCBs are also associated with adverse health outcomes (Carpenter 2006); however, because exposure is often to mixtures of PCBs, it is difficult to precisely ascertain which congener is responsible for a specific health outcome—some animal studies have attempted to fill in this gap (Johansen et al. 2011). In our study, there is an increased risk (*R*_95_ = 1.19 × 10^−6^) of exposure to PCB congener 153 from the consumption of goose for men (Fig. 2). It is a slight risk but does exceed the lifetime attributable risk (1.00 × 10^−6^).

Of the 23 samples of goose collected for this study, 16 had detectable limits of PCB congener 153 with a median of 4.50 × 10^−4^ mg/kg and a range of 9.00 × 10^−5^ to 5.40 × 10^−3^ mg/kg, which are higher values than a previous study (Tsuji et al. 2007b) carried out in a similar James Bay Cree community in Ontario, Canada (median: 1.00 × 10^−4^ mg/kg; maximum: 8.00 × 10^−4^ mg/kg). Furthermore, although we are unable to determine the different species of goose in our study, Tsuji et al. (2007b) confirm that seasonality influences contamination greatly among Canada geese (*Branta canadensis*), but not lesser snow geese (*Chen caerulescens caerulescens*), which may impact the extrapolation of our results.

Since the risk of exposure to PCB congener 153 is slight, we do not foresee goose consumption as a significant risk to the men who consume it in our study region. Men consume nearly twice as much goose as women (Table 2) and four times as much as children; therefore, we are not surprised that the sensitivity analysis showed that IR was a contributing factor to this risk because men consume more goose than the other demographics (Fig. 4; Table S7). Moreover, if this risk was a genuine concern, a slight reduction in consumption of approximately 3.5 g/day would be sufficient to bring the risk below the lifetime attributable risk of 1.00 × 10^−6^.

What may be of concern, however, is that the tissue concentration of PCBs seems to be increased in goose tissue compared to past studies (Braune and Malone 2006; Tsuji et al. 2007b), possibly indicating increasing PCB concentrations in geese in North America. However, we urge a cautionary view because we do not have specific harvesting locations, nor do we have specifics about the species of goose. Also, the sensitivity analysis showed that the concentration of the PCB congener 153 in the goose tissue was the greatest overall contributor to the attributable risk from consuming this traditional food. Therefore, we posit that additional study is required as previous work (Liberda et al. 2014) found that PCB congeners in blood were elevated in adults in this study region, but a route of exposure is not fully elucidated yet.

For comparison in other Indigenous communities in Canada and globally, traditional foods such as fish (e.g., arctic char) and marine mammals (e.g., whale) are contaminated with PCB congener 153—and other PCBs—and are the probable route of exposure, following consumption, to PCB congener 153 (Curren et al. 2015; Lakhmanov et al. 2020; Muckle et al. 2001; Singh et al. 2014). Consequently, there may be a risk to human health consuming these traditional foods, although most studies indicate the risk is slight, as does our study following the consumption of goose. Moreover, to our knowledge, there are no studies linking the consumption of goose, nor risk of exposure, to PCB congener 153. However, Tsuji et al. (2007b) found that PCB congener 153 was detected in breast tissue of Canada geese and lesser snow geese in a similar Indigenous region in Canada; thus, it is possible, but uncertain, that the consumption of goose could be a route of exposure to PCB congener 153. Nonetheless, further research is required.

#### PBB congener 153 exposure risk from duck consumption

The ducks in this study are contaminated with organohalogens, and PBB congener 153 seems to pose a risk to boys, women, and men as the probabilistic risk values 1.09 × 10^−6^, 1.57 × 10^−6^, and 1.19 × 10^−6^, respectively, exceed the lifetime attributable risk of 1.00 × 10^−6^ (Fig. 3). PBB congener 153 was typically used in fire retardants, foams, and plastics, and like PCB congener 153, it was banned from manufacture since the 1970s in the United States (Kodavanti and Loganathan 2014), but only since 1989 in Canada (Canada 2009b). However, PBB congener 153 does not persist in the environment in the same manner as PCB congener 153; it is typically more of a human health risk during acute, high-dose exposures, like those in accidental contaminations (Chang et al. 2020), although long-term epigenetic changes from chronic exposure to PBB congener 153 have been observed recently (Greeson et al. 2020).

Therefore, our findings suggest that these ducks were exposed to PBB congener 153 at some point in their life cycle; however, we are unable at the current time to elucidate when, where, how, or if the ducks’ exposure was chronic or acute.

There were seven duck samples used in our study, six of those had detectable limits of PBB congener 153, with a median value of 2.68 × 10^−3^ mg/kg and a range of 4.60 × 10^−4^ to 5.50 × 10^−3^ mg/kg. Boys, women, and men all had an *R*_95_ value greater than 1.00 × 10^−6^ for the consumption of duck as a traditional food (Table S6) using an oral slope factor of 8.90 (mg/kg/day)^−1^, and the sensitivity analysis demonstrated that the greatest contributor to this risk was the concentration of PBB congener 153 in the duck tissue (Fig. 4; Table S7). However, due to a paucity of studies with comparable information on traditional food consumption and/or the route of exposure to PBB congener 153 and other flame retardants, it is uncertain if these tissue concentrations pose an actual risk.

We further analyzed the data to elucidate why the traditional consumption of ducks led to an increased risk for some demographics in our study region. Following this reassessment, we noticed that three out of the available six duck samples had a range of 4.60 × 10^−3^ to 5.50 × 10^−3^ mg/kg, while the other three had a range of 4.60 × 10^−4^ to 6.70 × 10^−4^ mg/kg. The higher PBB congener 153 duck tissue concentrations came from fish-eating ducks, as the samples could be identified as such, rather than the more commonly consumed dabbling ducks in the study region. Fish is a known route of exposure to PBBs and other persistent organic compounds, whereas fish with higher fat is associated with increased PBB concentrations (Kodavanti and Loganathan 2014). Moreover, since the study region community members favor dabbling ducks because they have a less fishy taste and these ducks typically have lower tissue concentrations of PBB congener 153, we believe that this is not a genuine risk to those who consume ducks as a traditional food (Nieboer et al. 2013). However, a previous study (Liberda et al. 2014) in our study region found that PBB congener 153 was present in the blood plasma of women and men, but not boys; we therefore infer that given the frequent consumption of duck by women and men (Table 2) in our study region, there could *potentially* be an association between the consumption of duck and exposure to PBB congener 153, although this is entirely speculative, and we do not imply a route of exposure. Still, since we have no information on the specific duck species in our study, further research is required to ascertain if we are correct about risk and exposure and, more importantly, to investigate why some of our duck samples have such high tissue concentrations of organohalogens.

### Limitations

There were several limitations to our study that are important to highlight. First, we were unable to account or control for seasonality in the collection of the traditional foods, which can influence the level of contaminants. For example, Tsuji et al. (2007b) found that Canada geese sampled in the spring had PCB concentrations that were considerably higher than the fall harvested birds. We also experienced issues with the exact speciation of some traditional foods; thus, we had to group some of the foods under a global family name versus a specific species. For example, we could not differentiate between duck species other than fish-eating ducks and others. Additionally, we note that traditional foods are not consumed all year round as they are based on availability and seasonality, so the modeled risk may overestimate the real-world risk; this also limits the potential modeling of a bolus dose hazard or risk in this study. In addition, the chemical analyses were on raw tissue samples, and it is known that the cooking (or smoking) of the games’ meats may influence lipid levels and contaminant concentration in the cooked game meats. We also note that we could not account for the specific part of the traditional food being sampled (e.g., muscle tissue versus organ tissue) in all cases, and this could have skewed the contaminant levels when analyzed and/or limited our interpretation of the results. Furthermore, although we have successfully modeled the probabilistic risk of exposure to the contaminants in our study region, we have no human biomarkers to aid in confirmation of the route of exposure from traditional foods. Lastly, we note that all samples came from the Eeyou Istchee region and that some organisms, mainly fish, may show differing contaminant profiles within this area due to their location and life cycles. Despite these limitations, we are confident in our study’s novel and meaningful results that will promote further research.

## Conclusion

This study assessed the probabilistic exposure to contaminants from the consumption of traditional foods in Eeyou Istchee. As presented, most traditional foods did not appear to cause increased harm or risk of exposure to contaminants after consumption. Consuming goose may expose men to PCB congener 153, but goose consumption is typically seasonal. However, the consumption of duck may be of concern to *Eeyouch* boys, women, and men living in this region, as there appears to be a risk of exposure to PBB congener 153. Additionally, duck is a traditional food with a low risk of exposure to contaminants overall, but duck tissue samples from this region are highly contaminated with organohalogens (i.e., PCBs, PBBs, and pesticides) and further investigation is warranted. In the interim, the risks of eating this traditional food should be balanced with the benefits of a traditional diet. Additionally, Cree foods contain fats and vitamins that promote health as well as being culturally significant. Therefore, we must continue to work alongside and provide information to our community participants about how to make healthy dietary decisions.

## Supplementary information

Table S1 (DOCX 15 kb)

Table S2 (DOCX 16 kb)

Table S3 (DOCX 30 kb)

Table S4 (DOCX 27 kb)

Table S5 (DOCX 63 kb)

Table S6 (DOCX 56 kb)

Table S7 (DOCX 14 kb)

## Data Availability

Not applicable.

## References

[CR1] Adamou TY, Riva M, Muckle G, Laouan Sidi EA, Lemire M, Ayotte P (2020). Blood mercury and plasma polychlorinated biphenyls concentrations in pregnant Inuit women from Nunavik: temporal trends, 1992–2017. Sci Total Environ.

[CR2] Agus S, Akkaya H, Daglioglu N, Eyuboglu S, Atasayan O, Mete F, Colak C, Sandal S, Yilmaz B (2021) Polychlorinated biphenyls and organochlorine pesticides in breast milk samples and their correlation with dietary and reproductive factors in lactating mothers in Istanbul. Environ Sci Pollut Res. 10.1007/s11356-021-15863-6 10.1007/s11356-021-15863-6 34386925

[CR3] Akbar L, Zuk AM, Martin ID, Liberda EN, Tsuji LJS (2021). Potential obesogenic effect of a complex contaminant mixture on Cree First Nations adults of Northern Québec, Canada. Environ Res.

[CR4] Ali N, Shahzad K, Rashid MI, Shen H, Ismail IMI, Eqani SAMAS (2017). Currently used organophosphate and brominated flame retardants in the environment of China and other developing countries (2000–2016). Environ Sci Pollut Res.

[CR5] Aminov Z, Carpenter DO (2020). Serum concentrations of persistent organic pollutants and the metabolic syndrome in Akwesasne Mohawks, a Native American community. Environ Pollut.

[CR6] Aminov Z, Haase R, Rej R, Schymura MJ, Santiago-Rivera A, Morse G, DeCaprio A, Carpenter DO, null, null (2016). Diabetes prevalence in relation to serum concentrations of polychlorinated biphenyl (PCB) congener groups and three chlorinated pesticides in a Native American population. Environ Health Perspect.

[CR7] Barrio-Parra F, Dominguez-Castillo, with contributions from A. (2017) EnviroPRA: environmental probabilistic risk assessment tools

[CR8] Batal M, Johnson-Down L, Moubarac J-C, Ing A, Fediuk K, Sadik T, Chan HM, Willows N (2018). Sociodemographic associations of the dietary proportion of ultra-processed foods in First Nations peoples in the Canadian provinces of British Columbia, Manitoba, Alberta and Ontario. Int J Food Sci Nutr.

[CR9] Belinsky DL, Kuhnlein HV (2000). Macronutrient, mineral, and fatty acid composition of Canada goose (Branta canadensis): an important traditional food resource of the Eastern James Bay Cree of Quebec. J Food Compos Anal.

[CR10] Bimonte VM, Besharat ZM, Antonioni A, Cella V, Lenzi A, Ferretti E, Migliaccio S (2021). The endocrine disruptor cadmium: a new player in the pathophysiology of metabolic diseases. J Endocrinol Investig.

[CR11] Bordeleau S, Asselin H, Mazerolle MJ, Imbeau L (2016). “Is it still safe to eat traditional food?” Addressing traditional food safety concerns in aboriginal communities. Sci Total Environ.

[CR12] Boucher O, Muckle G, Saint-Amour D, Dewailly É, Ayotte P, Jacobson SW, Jacobson JL, Bastien CH (2009) The relation of lead neurotoxicity to the event-related potential P3b component in Inuit children from arctic Québec. NeuroToxicology, 10th International Symposium on Neurobehavioral Methods and Effects in Environmental and Occupational Health 30, 1070–1077. 10.1016/j.neuro.2009.06.008 10.1016/j.neuro.2009.06.008 PMC2789882 19576242

[CR13] Braune BM, Malone BJ (2006). Organochlorines and mercury in waterfowl harvested in Canada. Environ Monit Assess.

[CR14] Canada E , CC (2009a) Toxic substances list: PCBs [WWW Document]. URL https://www.canada.ca/en/environment-climate-change/services/management-toxic-substances/list-canadian-environmental-protection-act/polychlorinated-biphenyls.html. Accessed 13 Apr 2022

[CR15] Canada E, CC (2009b) Toxic substances list: polybrominated biphenyls [WWW Document]. URL https://www.canada.ca/en/environment-climate-change/services/management-toxic-substances/list-canadian-environmental-protection-act/polybrominated-biphenyls.html. Accessed 13 Mar 2022

[CR16] Canada H (2007) Eating well with Canada’s food guide - first nations, Inuit and Métis [WWW Document]. URL https://www.canada.ca/en/health-canada/services/food-nutrition/reports-publications/eating-well-canada-food-guide-first-nations-inuit-metis.html. Accessed 22 Oct 2021

[CR17] Carpenter D (2006). Polychlorinated biphenyls (PCBs): routes of exposure and effects on human health. Rev Environ Health.

[CR18] Carpenter DO (2014). Environmental exposure in indigenous communities: an international perspective. Rev Environ Health.

[CR19] Chan HM, Singh K, Batal M, Marushka L, Tikhonov C, Sadik T, Schwartz H, Ing A, Fediuk K (2021) Levels of metals and persistent organic pollutants in traditional foods consumed by First Nations living on-reserve in Canada. Can J Public Health :112:81–96. 10.17269/s41997-021-00495-7 10.17269/s41997-021-00495-7 PMC8239065 34181226

[CR20] Chang C-J, Terrell ML, Marcus M, Marder ME, Panuwet P, Ryan PB, Pearson M, Barton H, Barr DB (2020). Serum concentrations of polybrominated biphenyls (PBBs), polychlorinated biphenyls (PCBs) and polybrominated diphenyl ethers (PBDEs) in the Michigan PBB Registry 40 years after the PBB contamination incident. Environ Int.

[CR21] Charania NAS, Tsuji LJD, Martin IN, Liberda E, Coté S, Ayotte P, Dewailly E, Nieboer E (2014). An examination of traditional foods and cigarette smoking as cadmium sources among the nine First Nations of Eeyou Istchee, northern Quebec, Canada. Environ Sci Process Impacts.

[CR22] Curren MS, Liang CL, Davis K, Kandola K, Brewster J, Potyrala M, Chan HM (2015). Assessing determinants of maternal blood concentrations for persistent organic pollutants and metals in the eastern and western Canadian Arctic. Sci Total Environ.

[CR23] Delignette-Muller ML, Dutang C (2015). fitdistrplus: an R package for fitting distributions. J Stat Softw.

[CR24] Ebbesson SOE, Risica PM, Ebbesson LOE, Kennish JM, Tejero ME (2005). Omega-3 fatty acids improve glucose tolerance and components of the metabolic syndrome in Alaskan Eskimos: the Alaska Siberia project. Int J Circumpolar Health.

[CR25] Environmental Protection Agency (EPA) (2005) Human health risk assessment protocol: characterizing risk and hazard (chapter 7). U.S. Environmental Protection Agency, Office of solid waste and emergency response, Washington

[CR26] Erickson MD (2018). Analytical chemistry of PCBs.

[CR27] Fakhri Y, Mousavi Khaneghah A, Conti GO, Ferrante M, Khezri A, Darvishi A, Ahmadi M, Hasanzadeh V, Rahimizadeh A, Keramati H, Moradi B, Amanidaz N (2018). Probabilistic risk assessment (Monte Carlo simulation method) of Pb and Cd in the onion bulb (Allium cepa) and soil of Iran. Environ Sci Pollut Res.

[CR28] Gates A, Hanning RM, Gates M, Skinner K, Martin ID, Tsuji LJ (2012). Vegetable and fruit intakes of on-reserve First Nations schoolchildren compared to Canadian averages and current recommendations. Int J Environ Res Public Health.

[CR29] Gates A, Hanning RM, Gates M, Tsuji LJ (2016). The food and nutrient intakes of First Nations youth living in Northern Ontario, Canada: evaluation of a harvest sharing program. J Hunger Environ Nutr.

[CR30] Gaudin VL, Receveur O, Walz L, Girard F, Potvin L (2014). A mixed methods inquiry into the determinants of traditional food consumption among three Cree communities of Eeyou Istchee from an ecological perspective. Int J Circumpolar Health.

[CR31] Giroux J-F, Rodrigue J, Brook R, Patenaude-Monette M (2022) Canada goose populations harvested in Eastern James Bay by Eeyou Istchee Cree hunters. Avian Conserv Ecol 17. 10.5751/ACE-02059-170105

[CR32] Greeson KW, Fowler KL, Estave PM, Kate Thompson S, Wagner C, Clayton Edenfield R, Symosko KM, Steves AN, Marder EM, Terrell ML, Barton H, Koval M, Marcus M, Easley CA (2020). Detrimental effects of flame retardant, PBB153, exposure on sperm and future generations. Sci Rep.

[CR33] Horak K, Chipman R, Murphy L, Johnston J (2014). Environmental contaminant concentrations in Canada goose (Branta canadensis) muscle: probabilistic risk assessment for human consumers. J Food Prot.

[CR34] Huang Z, Qadeer A, Zheng S, Ge F, Zhang K, Yin D, Zheng B, Zhao X (2022). Fatty acid profile as an efficient bioindicator of PCB bioaccumulation in a freshwater lake food web: a stable isotope guided investigation. J Hazard Mater.

[CR35] Ijomone OM, Ijomone OK, Iroegbu JD, Ifenatuoha CW, Olung NF, Aschner M (2020). Epigenetic influence of environmentally neurotoxic metals. NeuroToxicology.

[CR36] Jeppesen C, Bjerregaard P, Jørgensen ME (2014). Dietary patterns in Greenland and their relationship with type 2 diabetes mellitus and glucose intolerance. Public Health Nutr.

[CR37] Jiménez-Oyola S, García-Martínez M-J, Ortega MF, Bolonio D, Rodríguez C, Esbrí J-M, Llamas JF, Higueras P (2020). Multi-pathway human exposure risk assessment using Bayesian modeling at the historically largest mercury mining district. Ecotoxicol Environ Saf.

[CR38] Johansen E, Knoff M, Fonnum F, Lausund P, Walaas S, Wøien G, Sagvolden T (2011). Postnatal exposure to PCB 153 and PCB 180, but not to PCB 52, produces changes in activity level and stimulus control in outbred male Wistar Kyoto rats. Behav Brain Funct.

[CR39] Juric AK, Batal M, David W, Sharp D, Schwartz H, Ing A, Fediuk K, Black A, Tikhonov C, Chan HM (2017). A total diet study and probabilistic assessment risk assessment of dietary mercury exposure among First Nations living on-reserve in Ontario, Canada. Environ Res.

[CR40] Juric AK, Batal M, David W, Sharp D, Schwartz H, Ing A, Fediuk K, Black A, Tikhonov C, Chan HM, Chan L (2018). Risk assessment of dietary lead exposure among First Nations people living on-reserve in Ontario, Canada using a total diet study and a probabilistic approach. J Hazard Mater.

[CR41] Kartalović B, Mastanjević K, Novakov N, Vranešević J, Ljubojević Pelić D, Puljić L, Habschied K (2020). Organochlorine pesticides and PCBs in traditionally and industrially smoked pork meat products from Bosnia and Herzegovina. Foods.

[CR42] Khare A, Jadhao P, Paliya S, Kumari K (2020) Toxicity and structural activity relationship of persistent organic pollutants. pp. 174–203. 10.2174/9789811460821120010012

[CR43] Kirchsteiger C (1999). On the use of probabilistic and deterministic methods in risk analysis. J Loss Prev Process Ind.

[CR44] Kodavanti P, Loganathan B (2014) Chapter 25. Polychlorinated biphenyls, polybrominated biphenyls, and brominated flame retardants. Biomark. Toxicol. 433–450. 10.1016/B978-0-12-404630-6.00025-7

[CR45] Koual M, Cano-Sancho G, Bats A-S, Tomkiewicz C, Kaddouch-Amar Y, Douay-Hauser N, Ngo C, Bonsang H, Deloménie M, Lecuru F, Le Bizec B, Marchand P, Botton J, Barouki R, Antignac J-P, Coumoul X (2019). Associations between persistent organic pollutants and risk of breast cancer metastasis. Environ Int.

[CR46] Kyllönen K, Vestenius M, Anttila P, Makkonen U, Aurela M, Wängberg I, Nerentorp Mastromonaco M, Hakola H (2020). Trends and source apportionment of atmospheric heavy metals at a subarctic site during 1996–2018. Atmos Environ.

[CR47] Lakhmanov D, Varakina Y, Aksenov A, Sorokina T, Sobolev N, Kotsur D, Plakhina E, Chashchin V, Thomassen Y (2020). Persistent organic pollutants (POPs) in fish consumed by the Indigenous peoples from Nenets Autonomous Okrug. Environments.

[CR48] Liberda EN, Tsuji LJS, Martin ID, Ayotte P, Robinson E, Dewailly E, Nieboer E (2018). Source identification of human exposure to lead in nine Cree Nations from Quebec, Canada (Eeyou Istchee territory). Environ Res.

[CR49] Liberda EN, Tsuji LJS, Martin ID, Cote S, Ayotte P, Dewailly E, Nieboer E (2014). Plasma concentrations of persistent organic pollutants in the Cree of northern Quebec, Canada: results from the multi-community environment-and-health study. Sci Total Environ.

[CR50] Liberda EN, Zuk AM, Di DS, Moriarity RJ, Martin ID, Tsuji LJS (2021). Complex environmental contaminant mixtures and their associations with thyroid hormones using supervised and unsupervised machine learning techniques. Environ Adv.

[CR51] Liberda EN, Zuk AM, Tsuji LJ (2019). Complex contaminant mixtures and their associations with intima-media thickness. BMC Cardiovasc Disord.

[CR52] Liberda EN, Zuk AM, Tsuji LJS (2021). Heart rate variation and human body burdens of environmental mixtures in the Cree First Nation communities of Eeyou Istchee, Canada. Environ Int.

[CR53] Lofstedt RE (2011). Risk versus hazard – how to regulate in the 21^st^ century. Eur J Risk Regul.

[CR54] Moriarity RJ, Liberda EN, Tsuji LJS (2020). Subsistence fishing in the Eeyou Istchee (James Bay, Quebec, Canada): a regional investigation of fish consumption as a route of exposure to methylmercury. Chemosphere.

[CR55] Moriarity RJ, Liberda EN, Tsuji LJS (2020b) Using a geographic information system to assess local scale methylmercury exposure in nine communities of the Eeyou Istchee territory (James Bay, Quebec, Canada). Environ. Res. 110147 10.1016/j.envres.2020.110147 32877705

[CR56] Muckle G, Ayotte P, Dewailly E, Jacobson SW, Jacobson JL, null (2001). Determinants of polychlorinated biphenyls and methylmercury exposure in inuit women of childbearing age. Environ Health Perspect.

[CR57] Munch-Andersen T, Olsen DB, Søndergaard H, Daugaard JR, Bysted A, Christensen DL, Saltin B, Helge JW (2012). Metabolic profile in two physically active Inuit groups consuming either a western or a traditional Inuit diet. Int J Circumpolar Health.

[CR58] Naqvi A, Qadir A, Mahmood A, Baqar M, Aslam I, Jamil N, Mumtaz M, Saeed S, Zhang G (2020). Screening of human health risk to infants associated with the polychlorinated biphenyl (PCB) levels in human milk from Punjab Province, Pakistan. Environ Sci Pollut Res.

[CR59] Nieboer ED, Martin IN, Liberda E, Dewailly E, Robinson ES, Tsuji LJ (2017). Body burdens, sources and interrelations of selected toxic and essential elements among the nine Cree First Nations of Eeyou Istchee, James Bay region of northern Quebec, Canada. Environ Sci Process Impacts.

[CR60] Nieboer Evert, Dewailly E, Johnson-Down L, Sampasa-Kanyinga H, Chateau-Degat M-L, Egeland GM, Atikesse L, Robinson E, Torrie J (2013) Nituuchischaayihtitaau Aschii multi-community environment-and-health study in Eeyou Istchee 2005-2009: final technical report. Appendix 1., in: Nieboer, E., Robinson, E., Petrov, K. (Eds.), Public health report series 4 on the health of the population. Cree Board of Health and Social Services of James Bay, Chisasibi, QC

[CR61] Noreen W, Johnson-Down L, Jean-Claude M, Lucas M, Robinson E, Batal M (2018). Factors associated with the intake of traditional foods in the Eeyou Istchee (Cree) of northern Quebec include age, speaking the Cree language and food sovereignty indicators. Int J Circumpolar Health.

[CR62] Öberg T, Bergbäck B (2005). A review of probabilistic risk assessment of contaminated land (12 pp). J Soils Sediments.

[CR63] Park EY, Park E, Kim J, Oh J-K, Kim B, Hong Y-C, Lim MK (2020). Impact of environmental exposure to persistent organic pollutants on lung cancer risk. Environ Int.

[CR64] Pelletier N, Chételat J, Palmer MJ, Vermaire JC (2021). Bog and lake sediment archives reveal a lagged response of subarctic lakes to diminishing atmospheric Hg and Pb deposition. Sci Total Environ.

[CR65] Pesiakova AA, Gusakova EV, Trofimova AN, Sorokina TY (2018). Migratory birds are the source of highly toxic organic pollutants for indigenous people in the Russian Arctic. IOP Conf Ser Earth Environ Sci.

[CR66] Popeski D, Ebbeling LR, Hornstra G (1991) Blood pressure during pregnancy in Canadian Inuit: community differences related to diet. CAN MED ASSOC J 10 PMC1335827 1878826

[CR67] R Core Team (2020) R: a language and environment for statistical computing

[CR68] Ramirez Prieto M, Ratelle M, Laird BD, Skinner K (2022). Dietary intakes of traditional foods for Dene/Métis in the Dehcho and Sahtú regions of the Northwest Territories. Nutrients.

[CR69] Ratelle M, Khoury C, Adlard B, Laird B (2020). Polycyclic aromatic hydrocarbons (PAHs) levels in urine samples collected in a subarctic region of the Northwest Territories, Canada. Environ Res.

[CR70] Ratelle M, Li XD, Laird B (2018). Cadmium exposure in First Nations communities of the Northwest Territories, Canada: smoking is a greater contributor than consumption of cadmium-accumulating organ meats. Environ Sci Process Impacts.

[CR71] Reyes ES, Liberda EN, Tsuji LJS (2015). Human exposure to soil contaminants in subarctic Ontario, Canada. Int J Circumpolar Health.

[CR72] Sanaei F, Amin MM, Alavijeh ZP, Esfahani RA, Sadeghi M, Bandarrig NS, Fatehizadeh A, Taheri E, Rezakazemi M (2021). Health risk assessment of potentially toxic elements intake via food crops consumption: Monte Carlo simulation-based probabilistic and heavy metal pollution index. Environ Sci Pollut Res.

[CR73] Sari MF, Esen F (2022) Concentration levels and an assessment of human health risk of polycyclic aromatic hydrocarbons (PAHs) and polychlorinated biphenyls (PCBs) in honey and pollen. Environ Sci Pollut Res. 10.1007/s11356-022-20545-y 10.1007/s11356-022-20545-y 35513623

[CR74] Singh A, Rajput P, Sharma D, Sarin MM, Singh D (2014). Black carbon and elemental carbon from postharvest agricultural-waste burning emissions in the Indo-Gangetic Plain. Adv Meteorol.

[CR75] Tchounwou PB, Yedjou CG, Patlolla AK, Sutton DJ (2012). Heavy metals toxicity and the environment. EXS.

[CR76] The Agency for Toxic Substances and Disease Registry (ATSDR) (2019) Appendix G: calculating exposure doses | PHA guidance manual | ATSDR [WWW Document]. URL https://www.atsdr.cdc.gov/hac/phamanual/appg.html. Accessed 22 Oct 2021

[CR77] Tomza-Marciniak A, Pilarczyk B, Witczak A, Rząd I, Pilarczyk R (2019). PCB residues in the tissues of sea ducks wintering on the south coast of the Baltic Sea, Poland. Environ Sci Pollut Res.

[CR78] Tsuji LJ, Nieboer E (1997). Lead pellet ingestion in First Nation Cree of the western James Bay region of northern Ontario, Canada: implications for a nontoxic shot alternative. Ecosyst Health.

[CR79] Tsuji LJ, Nieboer E, Karagatzides JD, Hanning RM, Katapatuk B (1999). Lead shot contamination in edible portions of game birds and its dietary implications. Ecosyst Health.

[CR80] Tsuji LJ, Wainman BC, Martin ID, Sutherland C, Weber J-P, Dumas P, Nieboer E (2008). Lead shot contribution to blood lead of First Nations people: the use of lead isotopes to identify the source of exposure. Sci Total Environ.

[CR81] Tsuji LJ, Wainman BC, Martin ID, Sutherland C, Weber J-P, Dumas P, Nieboer E (2008). The identification of lead ammunition as a source of lead exposure in First Nations: the use of lead isotope ratios. Sci Total Environ.

[CR82] Tsuji LJS, Manson H, Wainman BC, Vanspronsen EP, Shecapio-Blacksmith J, Rabbitskin T (2007). Identifying potential receptors and routes of contaminant exposure in the traditional territory of the Ouje-Bougoumou Cree: land use and a geographical information system. Environ Monit Assess.

[CR83] Tsuji LJS, Martin I, Martin E, Leblanc A, Dumas P (2007). Spring-harvested game birds from the western James Bay region of northern Ontario, Canada: organochlorine concentrations in breast muscle. Sci Total Environ.

[CR84] Tsuji LJS, Tsuji SRJ, Zuk AM, Davey R, Liberda EN (2020). Harvest programs in First Nations of subarctic Canada: the benefits go beyond addressing food security and environmental sustainability issues. Int J Environ Res Public Health.

[CR85] Tsuji LJS, Wainman BC, Jayasinghe RK, VanSpronsen EP, Liberda EN (2009). Determining tissue-lead levels in large game mammals harvested with lead bullets: human health concerns. Bull Environ Contam Toxicol.

[CR86] Urbano T, Filippini T, Lasagni D, De Luca T, Grill P, Sucato S, Polledri E, Djeukeu Noumbi G, Malavolti M, Santachiara A, Pertinhez TA, Baricchi R, Fustinoni S, Michalke B, Vinceti M (2021). Association of urinary and dietary selenium and of serum selenium species with serum alanine aminotransferase in a healthy Italian population. Antioxid Basel Switz.

[CR87] Urbano T, Filippini T, Wise LA, Lasagni D, De Luca T, Sucato S, Polledri E, Malavolti M, Rigon C, Santachiara A, Pertinhez TA, Baricchi R, Fustinoni S, Vinceti M (2022). Associations of urinary and dietary cadmium with urinary 8-oxo-7,8-dihydro-2’-deoxyguanosine and blood biochemical parameters. Environ Res.

[CR88] US Environmental Protection Agency (EPA), 2000. Assigning values to non-detected/non-quantified pesticide residues in human health exposure assessments

[CR89] US EPA (2015a) Regional guidance on handling chemical concentration data near the detection limit in risk assessments [WWW Document]. URL https://www.epa.gov/risk/regional-guidance-handling-chemical-concentration-data-near-detection-limit-risk-assessments . Accessed 22 Oct 2021

[CR90] US EPA (2015b) Use of Monte Carlo simulation in risk assessments [WWW Document]. URL: https://www.epa.gov/risk/use-monte-carlo-simulation-risk-assessments. Accessed 24 Nov 2021

[CR91] US EPA (2015c) Learn about polychlorinated biphenyls (PCBs) [WWW Document]. URL https://www.epa.gov/pcbs/learn-about-polychlorinated-biphenyls-pcbs. Accessed 13 Apr 2022

[CR92] Valera B, Dewailly É, Poirier P (2013). Association between methylmercury and cardiovascular risk factors in a native population of Quebec (Canada): a retrospective evaluation. Environ Res.

[CR93] Valera DE, Anassour-Laouan-Sidi E, Poirier P (2011a) Influence of n-3 fatty acids on cardiac autonomic activity among Nunavik Inuit adults. Int J Circumpolar Health 70:6–18. 10.3402/ijch.v70i1.17800 10.3402/ijch.v70i1.17800 21329579

[CR94] Valera DE, Dewailly E, Poirier P (2011b) Impact of mercury exposure on blood pressure and cardiac autonomic activity among Cree adults (James Bay, Quebec, Canada). Environ Res 111:1265–1270. 10.1016/j.envres.2011.09.001 10.1016/j.envres.2011.09.001 21962568

[CR95] Wainman BC, Kesner JS, Martin ID, Meadows JW, Krieg EF, Nieboer E, Tsuji LJ (2016). Menstrual cycle perturbation by organohalogens and elements in the Cree of James Bay, Canada. Chemosphere.

[CR96] Weitekamp CA, Shaffer RM, Chiang C, Lehmann GM, Christensen K (2022). An evidence map of polychlorinated biphenyl exposure and health outcome studies among residents of the Akwesasne Mohawk Nation. Chemosphere.

[CR97] Wickham H (2016) ggplot2: elegant graphics for data analysis

[CR98] Wong F, Shoeib M, Katsoyiannis A, Eckhardt S, Stohl A, Bohlin-Nizzetto P, Li H, Fellin P, Su Y, Hung H (2018). Assessing temporal trends and source regions of per- and polyfluoroalkyl substances (PFASs) in air under the Arctic Monitoring and Assessment Programme (AMAP). Atmos Environ.

[CR99] Wong SF, Lee BQ, Low KH, Jenatabadi HS, Wan Mohamed Radzi CWJB, Khor SM (2020). Estimation of the dietary intake and risk assessment of food carcinogens (3-MCPD and 1,3-DCP) in soy sauces by Monte Carlo simulation. Food Chem.

[CR100] Yilmaz B, Terekeci H, Sandal S, Kelestimur F (2020). Endocrine disrupting chemicals: exposure, effects on human health, mechanism of action, models for testing and strategies for prevention. Rev Endocr Metab Disord.

