# Supplementary material for: A probabilistic hazard and risk assessment of exposure to metals and organohalogens associated with a traditional diet in the Indigenous communities of Eeyou Istchee (northern Quebec, Canada)
Source: Environ Sci Pollut Res Int. 2022 Sep 24;30(6):14304–17. doi: 10.1007/s11356-022-23117-2 (PMC9908690; doi:10.1007/s11356-022-23117-2)
Supplement: Supplementary file 1 — (DOCX 15 kb) [file 11356_2022_23117_MOESM1_ESM.docx]

| **Contaminant** | **Reference dose**  **(mg/kg day)** | **Source** |
| --- | --- | --- |
| Al | 2.00 | https://apps.who.int/food-additives-contaminants-jecfa-database/chemical.aspx?chemID=298 |
| Sb | 0.0004 | IRIS (Integrated Risk Information System) |
| Ba | 0.20 | IRIS (Integrated Risk Information System) |
| Cd | 0.001 | HCTRV 2.0 (Health Canada Toxicological Reference Values) |
| Cr | 1.50 | IRIS (Integrated Risk Information System) |
| Cu (Adults) | 0.141 | HCTRV 2.0 (Health Canada Toxicological Reference Values) |
| Cu (Children) | 0.126 | HCTRV 2.0 (Health Canada Toxicological Reference Values) |
| Pb | 0.020 | https://apps.who.int/food-additives-contaminants-jecfa-database/chemical.aspx?chemID=3511 |
| Hg | 0.00047 | HCTRV 2.0 (Health Canada Toxicological Reference Values) |
| Mo | 28.00 | HCTRV 2.0 (Health Canada Toxicological Reference Values) |
| Ni | 0.011 | HCTRV 2.0 (Health Canada Toxicological Reference Values) |
| Se (Adults) | 5.70 | HCTRV 2.0 (Health Canada Toxicological Reference Values) |
| Se (Children) | 6.20 | HCTRV 2.0 (Health Canada Toxicological Reference Values) |
| Sn | 2.00 | https://apps.who.int/food-additives-contaminants-jecfa-database/chemical.aspx?chemID=515 |

**Table S1: Reference doses or slope factors for metals**

*Key*:

Al: Aluminum, An: Antimony, Ba: Barium, Cd: Cadmium, Cr: Chromium, Cu: Copper, Pb: Lead, Hg: Mercury, Mo: Molybdenum, Ni: Nickel, Se: Selenium, Sn: Tin
