# Supplementary material for: A probabilistic hazard and risk assessment of exposure to metals and organohalogens associated with a traditional diet in the Indigenous communities of Eeyou Istchee (northern Quebec, Canada)
Source: Environ Sci Pollut Res Int. 2022 Sep 24;30(6):14304–17. doi: 10.1007/s11356-022-23117-2 (PMC9908690; doi:10.1007/s11356-022-23117-2)
Supplement: Supplementary file 2 — (DOCX 16 kb) [file 11356_2022_23117_MOESM2_ESM.docx]

**Table S2: Reference doses or slope factors for organohalogens**

| **Contaminant** | **Reference dose**  **(mg/kg day)** | **Slope factor**  **(per mg/kg day)** | **Source** |
| --- | --- | --- | --- |
| Cis-nonachlor |  | 0.35 | IRIS (Integrated Risk Information System) |
| Mirex | 0.0002 |  | IRIS (Integrated Risk Information System) |
| Oxychlordane |  | 0.35 | https://www.gsi-net.com/en/publications/gsi-chemical-database/single/416-CAS-5103731.html |
| PBB, IUPAC # 153 |  | 8.90 | https://rais.ornl.gov/epa/heast/PolybrominatedBiphenyls.html |
| PBDE, IUPAC # 100 | 0.0001 |  | HCTRV 2.0 (Health Canada Toxicological Reference Values) |
| PBDE, IUPAC # 153 | 0.0001 |  | https://apps.who.int/food-additives-contaminants-jecfa-database/chemical.aspx?chemID=3511 |
| PBDE, IUPAC # 154 | 0.0001 |  | HCTRV 2.0 (Health Canada Toxicological Reference Values) |
| PBDE, IUPAC # 47 | 0.0001 |  | HCTRV 2.0 (Health Canada Toxicological Reference Values) |
| PCB, IUPAC # 101 |  | 2.00 | HCTRV 2.0 (Health Canada Toxicological Reference Values) |
| PCB, IUPAC # 118 |  | 2.00 | HCTRV 2.0 (Health Canada Toxicological Reference Values) |
| PCB, IUPAC # 128 |  | 2.00 | https://www.gsi-net.com/en/publications/gsi-chemical-database/single/416-CAS-5103731.html |
| PCB, IUPAC # 138 |  | 2.00 | https://apps.who.int/food-additives-contaminants-jecfa-database/chemical.aspx?chemID=515 |
| PCB, IUPAC # 153 |  | 2.00 | HCTRV 2.0 (Health Canada Toxicological Reference Values) |
| PCB, IUPAC # 156 |  | 2.00 | https://www.gsi-net.com/en/publications/gsi-chemical-database/single/416-CAS-5103731.html |
| PCB, IUPAC # 163 |  | 2.00 | https://www.gsi-net.com/en/publications/gsi-chemical-database/single/508-thallium-as-thallium-chloride.html |
| PCB, IUPAC # 170 |  | 2.00 | HCTRV 2.0 (Health Canada Toxicological Reference Values) |
| PCB, IUPAC # 180 |  | 2.00 | HCTRV 2.0 (Health Canada Toxicological Reference Values) |
| PCB, IUPAC # 183 |  | 2.00 | HCTRV 2.0 (Health Canada Toxicological Reference Values) |
| PCB, IUPAC # 187 |  | 2.00 | HCTRV 2.0 (Health Canada Toxicological Reference Values) |
| PCB, IUPAC # 194 |  | 2.00 | HCTRV 2.0 (Health Canada Toxicological Reference Values) |
| PCB, IUPAC # 28 |  | 2.00 | HCTRV 2.0 (Health Canada Toxicological Reference Values) |
| PCB, IUPAC # 52 |  | 2.00 | HCTRV 2.0 (Health Canada Toxicological Reference Values) |
| PCB, IUPAC # 99 |  | 2.00 | HCTRV 2.0 (Health Canada Toxicological Reference Values) |
| p,p'-DDD |  | 0.24 | IRIS (Integrated Risk Information System) |
| p,p'-DDE |  | 0.34 | IRIS (Integrated Risk Information System) |
| p,p'-DDT |  | 0.34 | IRIS (Integrated Risk Information System) |
| β-Hexachlorocyclohexane |  | 1.80 | IRIS (Integrated Risk Information System) |
| Toxaphene, Parlar no. 26 |  | 1.10 | IRIS (Integrated Risk Information System) |
| Toxaphene, Parlar no. 50 |  | 1.10 | IRIS (Integrated Risk Information System) |
| Trans-nonachlor |  | 0.35 | https://www.gsi-net.com/en/publications/gsi-chemical-database/single/416-CAS-5103731.html |

*Key*:

PCB: polychlorinated biphenyl, PBB: polybrominated biphenyl, PBDE: polybrominated diphenyl ethers, DDD: dichlorodiphenyldichloroethane, DDE: dichlorodiphenyldichloroethylene, DDT: dichlorodiphenyltrichloroethane
