# Supplementary material for: A probabilistic hazard and risk assessment of exposure to metals and organohalogens associated with a traditional diet in the Indigenous communities of Eeyou Istchee (northern Quebec, Canada)
Source: Environ Sci Pollut Res Int. 2022 Sep 24;30(6):14304–17. doi: 10.1007/s11356-022-23117-2 (PMC9908690; doi:10.1007/s11356-022-23117-2)
Supplement: Supplementary file 3 — (DOCX 30 kb) [file 11356_2022_23117_MOESM3_ESM.docx]

**Table S3: Descriptive statistics of metals by traditional food included in the hazard or risk examinations**

| **Species** | **Contaminant** | ***n***  **>MLOD** | **Concentration (mg/kg)** | | | | | | | | | |
| --- | --- | --- | --- | --- | --- | --- | --- | --- | --- | --- | --- | --- |
|  |  |  | **Mean** | ***s*** | **min** | **P_5_** | **P_25_** | **P_50_** | **P_75_** | **P_95_** | **P_99_** | **max** |
| Bear | Al | 14 | 2.34 | 2.42 | 0.80 | 0.86 | 1.00 | 1.00 | 2.65 | 7.70 | 7.70 | 7.70 |
| (n = 15) | Ba | 14 | 0.05 | 0.03 | 0.01 | 0.02 | 0.02 | 0.04 | 0.08 | 0.09 | 0.10 | 0.10 |
|  | Cd | 14 | 0.04 | 0.03 | 0.01 | 0.01 | 0.02 | 0.03 | 0.07 | 0.10 | 0.10 | 0.10 |
|  | Cr | 8 | 0.12 | 0.05 | 0.05 | 0.06 | 0.09 | 0.12 | 0.15 | 0.20 | 0.20 | 0.20 |
|  | Cu | 14 | 1.89 | 0.36 | 1.20 | 1.46 | 1.80 | 1.80 | 2.05 | 2.50 | 2.66 | 2.70 |
|  | Pb | 13 | 0.03 | 0.04 | 0.00 | 0.00 | 0.00 | 0.01 | 0.03 | 0.11 | 0.11 | 0.11 |
|  | Hg | 10 | 0.03 | 0.03 | 0.01 | 0.01 | 0.01 | 0.01 | 0.06 | 0.06 | 0.06 | 0.06 |
|  | Mo | 14 | 0.02 | 0.01 | 0.01 | 0.01 | 0.01 | 0.02 | 0.02 | 0.04 | 0.05 | 0.05 |
|  | Ni | 10 | 0.03 | 0.01 | 0.02 | 0.02 | 0.02 | 0.03 | 0.04 | 0.04 | 0.04 | 0.04 |
|  | Se | 14 | 0.22 | 0.07 | 0.10 | 0.10 | 0.20 | 0.21 | 0.28 | 0.32 | 0.32 | 0.32 |
|  |  |  |  |  |  |  |  |  |  |  |  |  |
| Beaver | Al | 14 | 0.00 | 0.00 | 0.00 | 0.00 | 0.00 | 0.00 | 0.00 | 0.00 | 0.00 | 0.00 |
| (n = 22) | Cd | 17 | 0.26 | 0.49 | 0.04 | 0.05 | 0.06 | 0.09 | 0.20 | 1.02 | 1.64 | 1.80 |
|  | Cr | 12 | 0.07 | - | 0.07 | 0.07 | 0.07 | 0.07 | 0.07 | 0.07 | 0.07 | 0.07 |
|  | Cu | 20 | 0.04 | 0.09 | 0.00 | 0.00 | 0.00 | 0.00 | 0.01 | 0.20 | 0.30 | 0.33 |
|  | Pb | 15 | 0.88 | 1.46 | 0.06 | 0.06 | 0.10 | 0.22 | 1.10 | 3.17 | 5.03 | 5.50 |
|  | Hg | 12 | 0.04 | 0.04 | 0.01 | 0.01 | 0.01 | 0.03 | 0.08 | 0.08 | 0.08 | 0.08 |
|  |  |  |  |  |  |  |  |  |  |  |  |  |
| Caribou | Ba | 5 | 0.07 | 0.04 | 0.03 | 0.03 | 0.03 | 0.08 | 0.08 | 0.12 | 0.13 | 0.13 |
| (n = 5) | Cd | 5 | 0.01 | 0.00 | 0.01 | 0.01 | 0.01 | 0.01 | 0.01 | 0.01 | 0.01 | 0.01 |
|  | Cu | 5 | 3.10 | 0.92 | 2.10 | 2.10 | 2.10 | 3.70 | 3.70 | 3.86 | 3.89 | 3.90 |
|  | Pb | 4 | 0.01 | 0.00 | 0.00 | 0.00 | 0.00 | 0.01 | 0.01 | 0.01 | 0.01 | 0.01 |
|  | Hg | 4 | 0.01 | 0.00 | 0.01 | 0.01 | 0.01 | 0.01 | 0.01 | 0.01 | 0.01 | 0.01 |
|  | Se | 5 | 0.46 | 0.15 | 0.20 | 0.26 | 0.50 | 0.50 | 0.55 | 0.55 | 0.55 | 0.55 |
|  |  |  |  |  |  |  |  |  |  |  |  |  |
| Duck | Al | 5 | 1.14 | 0.54 | 0.80 | 0.82 | 0.90 | 0.90 | 1.00 | 1.88 | 2.06 | 2.10 |
| (n = 7) | Ba | 5 | 0.16 | 0.18 | 0.01 | 0.02 | 0.04 | 0.05 | 0.36 | 0.36 | 0.36 | 0.36 |
|  | Cd | 6 | 0.03 | 0.02 | 0.00 | 0.00 | 0.01 | 0.03 | 0.05 | 0.05 | 0.05 | 0.05 |
|  | Cr | 4 | 0.52 | 0.49 | 0.07 | 0.08 | 0.11 | 0.53 | 0.94 | 0.94 | 0.94 | 0.94 |
|  | Cu | 7 | 4.30 | 2.47 | 2.50 | 2.59 | 2.85 | 2.90 | 5.55 | 7.90 | 7.90 | 7.90 |
|  | Pb | 6 | 0.01 | 0.01 | 0.00 | 0.00 | 0.01 | 0.01 | 0.01 | 0.02 | 0.02 | 0.02 |
|  | Hg | 6 | 0.57 | 0.57 | 0.09 | 0.11 | 0.20 | 0.28 | 1.05 | 1.30 | 1.30 | 1.30 |
|  | Mo | 6 | 0.02 | 0.01 | 0.01 | 0.01 | 0.01 | 0.02 | 0.03 | 0.03 | 0.03 | 0.03 |
|  | Se | 6 | 1.65 | 1.08 | 0.66 | 0.66 | 0.90 | 1.60 | 1.75 | 3.15 | 3.51 | 3.60 |
|  |  |  |  |  |  |  |  |  |  |  |  |  |
| Goose | Sb | 12 | 0.02 | 0.03 | 0.00 | 0.00 | 0.00 | 0.00 | 0.01 | 0.06 | 0.09 | 0.10 |
| (n = 23) | Ba | 17 | 0.04 | 0.03 | 0.00 | 0.00 | 0.01 | 0.03 | 0.04 | 0.10 | 0.10 | 0.10 |
|  | Cr | 10 | 0.13 | 0.13 | 0.03 | 0.03 | 0.05 | 0.06 | 0.19 | 0.36 | 0.38 | 0.38 |
|  | Cu | 19 | 4.14 | 1.57 | 1.30 | 1.39 | 3.15 | 4.60 | 5.30 | 6.34 | 6.63 | 6.70 |
|  | Pb | 13 | 0.15 | 0.47 | 0.00 | 0.00 | 0.01 | 0.02 | 0.04 | 0.74 | 1.51 | 1.70 |
|  | Mo | 19 | 0.02 | 0.01 | 0.01 | 0.01 | 0.01 | 0.02 | 0.02 | 0.03 | 0.04 | 0.04 |
|  | Se | 19 | 0.20 | 0.06 | 0.10 | 0.10 | 0.18 | 0.20 | 0.24 | 0.28 | 0.30 | 0.30 |
|  |  |  |  |  |  |  |  |  |  |  |  |  |
| Grouse | Ba | 9 | 0.15 | 0.17 | 0.01 | 0.01 | 0.03 | 0.08 | 0.13 | 0.45 | 0.50 | 0.51 |
| (n = 13) | Cd | 10 | 0.02 | 0.02 | 0.00 | 0.00 | 0.00 | 0.01 | 0.03 | 0.06 | 0.06 | 0.06 |
|  | Cu | 12 | 1.99 | 1.02 | 0.68 | 0.91 | 1.25 | 1.65 | 2.80 | 3.58 | 3.76 | 3.80 |
|  | Pb | 11 | 0.30 | 0.77 | 0.00 | 0.00 | 0.01 | 0.01 | 0.18 | 1.45 | 2.37 | 2.60 |
|  | Mo | 9 | 0.02 | 0.01 | 0.01 | 0.01 | 0.01 | 0.01 | 0.02 | 0.03 | 0.04 | 0.04 |
|  | Se | 12 | 0.29 | 0.11 | 0.07 | 0.13 | 0.24 | 0.30 | 0.33 | 0.44 | 0.46 | 0.46 |
|  | Sn | 10 | 0.04 | 0.00 | 0.04 | 0.04 | 0.04 | 0.04 | 0.04 | 0.04 | 0.04 | 0.04 |
|  |  |  |  |  |  |  |  |  |  |  |  |  |
| Hare | Ba | 16 | 0.51 | 1.63 | 0.01 | 0.01 | 0.02 | 0.04 | 0.21 | 1.94 | 5.67 | 6.60 |
| (n = 19) | Cd | 17 | 0.01 | 0.02 | 0.00 | 0.00 | 0.01 | 0.01 | 0.01 | 0.03 | 0.08 | 0.10 |
|  | Cr | 11 | 0.19 | 0.37 | 0.03 | 0.04 | 0.05 | 0.07 | 0.12 | 0.74 | 1.19 | 1.30 |
|  | Cu | 17 | 2.40 | 0.42 | 1.90 | 1.90 | 2.10 | 2.20 | 2.70 | 3.10 | 3.10 | 3.10 |
|  | Se | 16 | 0.14 | 0.09 | 0.06 | 0.06 | 0.07 | 0.10 | 0.20 | 0.30 | 0.33 | 0.34 |
|  |  |  |  |  |  |  |  |  |  |  |  |  |
| Moose | Al | 20 | 29.09 | 113.27 | 0.50 | 0.60 | 0.88 | 2.05 | 6.00 | 40.70 | 416.14 | 510.00 |
| (n = 37) | Ba | 35 | 0.27 | 0.81 | 0.00 | 0.01 | 0.03 | 0.05 | 0.18 | 0.67 | 3.45 | 4.80 |
|  | Cd | 34 | 0.18 | 0.92 | 0.00 | 0.00 | 0.01 | 0.01 | 0.02 | 0.09 | 3.64 | 5.30 |
|  | Cr | 23 | 0.58 | 1.22 | 0.03 | 0.04 | 0.05 | 0.12 | 0.38 | 1.97 | 4.81 | 5.60 |
|  | Cu | 35 | 12.16 | 62.27 | 0.20 | 0.75 | 1.25 | 1.60 | 1.90 | 3.33 | 245.83 | 370.00 |
|  | Pb | 21 | 0.08 | 0.26 | 0.00 | 0.00 | 0.01 | 0.01 | 0.03 | 0.14 | 0.99 | 1.20 |
|  | Se | 29 | 0.12 | 0.17 | 0.05 | 0.05 | 0.07 | 0.08 | 0.10 | 0.25 | 0.80 | 1.00 |
|  |  |  |  |  |  |  |  |  |  |  |  |  |
| Walleye | Ba | 6 | 0.03 | 0.02 | 0.01 | 0.01 | 0.01 | 0.02 | 0.03 | 0.05 | 0.06 | 0.06 |
| (n = 10) | Cu | 10 | 0.33 | 0.09 | 0.20 | 0.22 | 0.30 | 0.32 | 0.36 | 0.46 | 0.51 | 0.52 |
|  | Pb | 9 | 0.00 | 0.00 | 0.00 | 0.00 | 0.00 | 0.00 | 0.00 | 0.00 | 0.00 | 0.00 |
|  | Hg | 10 | 0.67 | 0.26 | 0.46 | 0.47 | 0.49 | 0.58 | 0.66 | 1.15 | 1.19 | 1.20 |
|  | Se | 10 | 0.43 | 0.06 | 0.30 | 0.33 | 0.42 | 0.44 | 0.47 | 0.51 | 0.53 | 0.53 |
|  | Sn | 9 | 0.04 | 0.00 | 0.04 | 0.04 | 0.04 | 0.04 | 0.04 | 0.04 | 0.04 | 0.04 |

*Key*:

MLOD: minimum level of detection, P*_n_*: *n^t^*^h^-percentile, *s*: standard deviation; Al: Aluminum, An: Antimony, Ba: Barium, Cd: Cadmium, Cr: Chromium, Cu: Copper, Pb: Lead, Hg: Mercury, Mo: Molybdenum, Ni: Nickel, Se: Selenium, Sn: Tin.
