# Supplementary material for: A probabilistic hazard and risk assessment of exposure to metals and organohalogens associated with a traditional diet in the Indigenous communities of Eeyou Istchee (northern Quebec, Canada)
Source: Environ Sci Pollut Res Int. 2022 Sep 24;30(6):14304–17. doi: 10.1007/s11356-022-23117-2 (PMC9908690; doi:10.1007/s11356-022-23117-2)
Supplement: Supplementary file 4 — (DOCX 27 kb) [file 11356_2022_23117_MOESM4_ESM.docx]

**Table S4: Descriptive statistics of organohalogens by traditional food included in the hazard or risk examinations**

| **Species** | **Contaminant** | ***n***  **>MLOD** | **Concentration (mg/kg)** | | | | | | | | | |
| --- | --- | --- | --- | --- | --- | --- | --- | --- | --- | --- | --- | --- |
|  |  |  | **Mean** | ***s*** | **min** | **P_5_** | **P_25_** | **P_50_** | **P_75_** | **P_95_** | **P_99_** | **max** |
| Bear | PCB, IUPAC # 153 | 8 | 0.0006 | 0.0010 | 0.0001 | 0.0001 | 0.0002 | 0.0002 | 0.0004 | 0.0021 | 0.0028 | 0.0030 |
| (n = 15) | PCB, IUPAC # 170 | 9 | 0.0003 | 0.0004 | 0.0001 | 0.0001 | 0.0001 | 0.0001 | 0.0002 | 0.0009 | 0.0012 | 0.0013 |
|  | PCB, IUPAC # 180 | 10 | 0.0005 | 0.0007 | 0.0002 | 0.0002 | 0.0003 | 0.0003 | 0.0005 | 0.0016 | 0.0022 | 0.0024 |
|  | PCB, IUPAC # 52 | 8 | 0.0002 | 0.0002 | 0.0002 | 0.0002 | 0.0002 | 0.0002 | 0.0000 | 0.0000 | 0.0000 | 0.0000 |
|  |  |  |  |  |  |  |  |  |  |  |  |  |
| Duck | Cis-nonachlor | 6 | 0.0068 | 0.0134 | 0.0003 | 0.0003 | 0.0006 | 0.0015 | 0.0026 | 0.0263 | 0.0325 | 0.0340 |
| (n =7) | Mirex | 6 | 0.0348 | 0.0386 | 0.0090 | 0.0090 | 0.0100 | 0.0235 | 0.0340 | 0.0910 | 0.1062 | 0.1100 |
|  | Oxychlordane | 6 | 0.0216 | 0.0237 | 0.0038 | 0.0038 | 0.0056 | 0.0165 | 0.0220 | 0.0558 | 0.0648 | 0.0670 |
|  | PBB, IUPAC # 153 | 6 | 0.0029 | 0.0026 | 0.0005 | 0.0005 | 0.0005 | 0.0027 | 0.0053 | 0.0055 | 0.0055 | 0.0055 |
|  | PBDE, IUPAC # 100 | 6 | 0.0262 | 0.0331 | 0.0017 | 0.0017 | 0.0027 | 0.0178 | 0.0300 | 0.0735 | 0.0851 | 0.0880 |
|  | PBDE, IUPAC # 153 | 6 | 0.0217 | 0.0204 | 0.0027 | 0.0027 | 0.0032 | 0.0208 | 0.0370 | 0.0438 | 0.0456 | 0.0460 |
|  | PBDE, IUPAC # 154 | 4 | 0.0073 | 0.0066 | 0.0015 | 0.0015 | 0.0015 | 0.0073 | 0.0130 | 0.0130 | 0.0130 | 0.0130 |
|  | PBDE, IUPAC # 47 | 6 | 0.0177 | 0.0384 | 0.0011 | 0.0011 | 0.0011 | 0.0011 | 0.0047 | 0.0735 | 0.0915 | 0.0960 |
|  | PCB, IUPAC # 101 | 6 | 0.0119 | 0.0231 | 0.0008 | 0.0008 | 0.0014 | 0.0032 | 0.0042 | 0.0454 | 0.0563 | 0.0590 |
|  | PCB, IUPAC # 118 | 6 | 0.2065 | 0.1934 | 0.0310 | 0.0310 | 0.0350 | 0.1885 | 0.3300 | 0.4350 | 0.4630 | 0.4700 |
|  | PCB, IUPAC # 128 | 6 | 0.0692 | 0.0734 | 0.0075 | 0.0075 | 0.0091 | 0.0560 | 0.0980 | 0.1670 | 0.1854 | 0.1900 |
|  | PCB, IUPAC # 138 | 6 | 0.3178 | 0.3408 | 0.0310 | 0.0310 | 0.0420 | 0.2575 | 0.4400 | 0.7775 | 0.8675 | 0.8900 |
|  | PCB, IUPAC # 153 | 6 | 0.5580 | 0.5308 | 0.0540 | 0.0540 | 0.0855 | 0.5300 | 0.8800 | 1.1950 | 1.2790 | 1.3000 |
|  | PCB, IUPAC # 156 | 6 | 0.0371 | 0.0351 | 0.0049 | 0.0049 | 0.0051 | 0.0359 | 0.0660 | 0.0728 | 0.0746 | 0.0750 |
|  | PCB, IUPAC # 163 | 6 | 0.0668 | 0.0848 | 0.0064 | 0.0064 | 0.0103 | 0.0450 | 0.0680 | 0.1895 | 0.2219 | 0.2300 |
|  | PCB, IUPAC # 170 | 6 | 0.1175 | 0.1160 | 0.0110 | 0.0110 | 0.0115 | 0.1165 | 0.2200 | 0.2275 | 0.2295 | 0.2300 |
|  | PCB, IUPAC # 180 | 6 | 0.3793 | 0.3732 | 0.0330 | 0.0330 | 0.0373 | 0.3850 | 0.7200 | 0.7200 | 0.7200 | 0.7200 |
|  | PCB, IUPAC # 183 | 6 | 0.0859 | 0.0843 | 0.0063 | 0.0063 | 0.0105 | 0.0815 | 0.1400 | 0.1850 | 0.1970 | 0.2000 |
|  | PCB, IUPAC # 187 | 6 | 0.2060 | 0.1984 | 0.0140 | 0.0140 | 0.0300 | 0.1990 | 0.3200 | 0.4475 | 0.4815 | 0.4900 |
|  | PCB, IUPAC # 194 | 4 | 0.0048 | 0.0022 | 0.0029 | 0.0029 | 0.0029 | 0.0048 | 0.0067 | 0.0067 | 0.0067 | 0.0067 |
|  | PCB, IUPAC # 28 | 6 | 0.0631 | 0.0635 | 0.0038 | 0.0064 | 0.0140 | 0.0405 | 0.1218 | 0.1400 | 0.1400 | 0.1400 |
|  | PCB, IUPAC # 52 | 4 | 0.0933 | 0.1002 | 0.0065 | 0.0065 | 0.0065 | 0.0933 | 0.1800 | 0.1800 | 0.1800 | 0.1800 |
|  | PCB, IUPAC # 99 | 6 | 0.0773 | 0.1169 | 0.0025 | 0.0025 | 0.0091 | 0.0445 | 0.0600 | 0.2475 | 0.2975 | 0.3100 |
|  | p,p'-DDD | 6 | 0.0062 | 0.0112 | 0.0009 | 0.0009 | 0.0011 | 0.0015 | 0.0027 | 0.0225 | 0.0277 | 0.0290 |
|  | p,p'-DDE | 6 | 0.5507 | 0.5121 | 0.0520 | 0.0520 | 0.0990 | 0.5350 | 0.8300 | 1.1825 | 1.2765 | 1.3000 |
|  | p,p'-DDT | 6 | 0.0101 | 0.0211 | 0.0003 | 0.0003 | 0.0005 | 0.0012 | 0.0038 | 0.0409 | 0.0506 | 0.0530 |
|  | β-HCH | 6 | 0.0014 | 0.0016 | 0.0004 | 0.0004 | 0.0004 | 0.0004 | 0.0024 | 0.0036 | 0.0038 | 0.0038 |
|  | Toxaphene, Parlar no. 26 | 4 | 0.0026 | 0.0010 | 0.0018 | 0.0018 | 0.0018 | 0.0023 | 0.0030 | 0.0038 | 0.0040 | 0.0040 |
|  | Toxaphene, Parlar no. 50 | 4 | 0.0026 | 0.0010 | 0.0018 | 0.0018 | 0.0018 | 0.0023 | 0.0030 | 0.0038 | 0.0040 | 0.0040 |
|  | Trans-nonachlor | 6 | 0.0060 | 0.0098 | 0.0016 | 0.0016 | 0.0017 | 0.0021 | 0.0023 | 0.0201 | 0.0248 | 0.0260 |
|  |  |  |  |  |  |  |  |  |  |  |  |  |
| Goose | PCB, IUPAC # 153 | 16 | 0.0009 | 0.0013 | 0.0001 | 0.0001 | 0.0002 | 0.0005 | 0.0011 | 0.0031 | 0.0049 | 0.0054 |
| (n = 23) | p,p'-DDE | 14 | 0.0224 | 0.0310 | 0.0020 | 0.0027 | 0.0046 | 0.0090 | 0.0235 | 0.0782 | 0.1036 | 0.1100 |
|  |  |  |  |  |  |  |  |  |  |  |  |  |
| Walleye | Cis-nonachlor | 5 | 0.0002 | 0.0001 | 0.0001 | 0.0001 | 0.0001 | 0.0002 | 0.0002 | 0.0004 | 0.0004 | 0.0004 |
| (n = 10) | PBDE, IUPAC # 100 | 6 | 0.0002 | 0.0000 | 0.0002 | 0.0002 | 0.0002 | 0.0002 | 0.0002 | 0.0002 | 0.0002 | 0.0002 |
|  | PBDE, IUPAC # 47 | 8 | 0.0004 | 0.0004 | 0.0001 | 0.0002 | 0.0003 | 0.0003 | 0.0004 | 0.0010 | 0.0012 | 0.0013 |
|  | PBDE, IUPAC # 99 | 7 | 0.0005 | 0.0002 | 0.0003 | 0.0003 | 0.0003 | 0.0004 | 0.0005 | 0.0009 | 0.0010 | 0.0010 |
|  | PCB, IUPAC # 118 | 7 | 0.0002 | 0.0001 | 0.0001 | 0.0001 | 0.0001 | 0.0002 | 0.0002 | 0.0003 | 0.0003 | 0.0003 |
|  | PCB, IUPAC # 128 | 7 | 0.0001 | 0.0000 | 0.0001 | 0.0001 | 0.0001 | 0.0001 | 0.0001 | 0.0002 | 0.0002 | 0.0002 |
|  | PCB, IUPAC # 138 | 9 | 0.0004 | 0.0002 | 0.0001 | 0.0001 | 0.0003 | 0.0004 | 0.0004 | 0.0006 | 0.0007 | 0.0007 |
|  | PCB, IUPAC # 153 | 10 | 0.0004 | 0.0003 | 0.0001 | 0.0001 | 0.0003 | 0.0005 | 0.0005 | 0.0009 | 0.0009 | 0.0010 |
|  | PCB, IUPAC # 170 | 7 | 0.0001 | 0.0000 | 0.0001 | 0.0001 | 0.0001 | 0.0001 | 0.0001 | 0.0002 | 0.0002 | 0.0002 |
|  | PCB, IUPAC # 180 | 9 | 0.0003 | 0.0001 | 0.0001 | 0.0001 | 0.0003 | 0.0003 | 0.0004 | 0.0005 | 0.0005 | 0.0005 |
|  | PCB, IUPAC # 187 | 9 | 0.0003 | 0.0001 | 0.0001 | 0.0001 | 0.0002 | 0.0003 | 0.0003 | 0.0004 | 0.0004 | 0.0004 |
|  | p,p'-DDE | 7 | 0.0014 | 0.0011 | 0.0007 | 0.0007 | 0.0008 | 0.0010 | 0.0015 | 0.0031 | 0.0036 | 0.0037 |

*Key*:

MLOD: minimum level of detection, P*_n_*: *n*^th^-percentile, *s*: standard deviation; PCB: polychlorinated biphenyl, PBB: polybrominated biphenyl, PBDE: polybrominated diphenyl ethers, DDD: dichlorodiphenyldichloroethane, DDE: dichlorodiphenyldichloroethylene, DDT: dichlorodiphenyltrichloroethane; HCH: Hexachlorocyclohexane
