# Supplementary material for: A probabilistic hazard and risk assessment of exposure to metals and organohalogens associated with a traditional diet in the Indigenous communities of Eeyou Istchee (northern Quebec, Canada)
Source: Environ Sci Pollut Res Int. 2022 Sep 24;30(6):14304–17. doi: 10.1007/s11356-022-23117-2 (PMC9908690; doi:10.1007/s11356-022-23117-2)
Supplement: Supplementary file 5 — (DOCX 63 kb) [file 11356_2022_23117_MOESM5_ESM.docx]

**Table S5: Probabilistic hazard for metal contaminants for traditional foods at n > 5 and minimum level of detection > 50% of n of contaminant**

| **Species** | **Demographic** | **Contaminant** | **P_5_** | **P_25_** | **P_50_** | **P_75_** | **P_95_** | **P_99_** |
| --- | --- | --- | --- | --- | --- | --- | --- | --- |
| Bear | Girls | Al | 2.84E-06 | 7.01E-06 | 1.11E-05 | 1.64E-05 | 3.08E-05 | 8.20E-05 |
|  |  | Ba | 4.66E-24 | 4.31E-09 | 1.63E-04 | 2.42E-04 | 4.51E-04 | 1.33E-03 |
|  |  | Cd | 9.08E-26 | 3.37E-08 | 2.90E-02 | 4.76E-02 | 8.96E-02 | 1.37E-01 |
|  |  | Cr | 3.33E-13 | 3.15E-06 | 2.50E-05 | 3.40E-05 | 6.28E-05 | 1.96E-04 |
|  |  | Cu | 7.73E-05 | 1.83E-04 | 2.56E-04 | 3.45E-04 | 6.23E-04 | 1.92E-03 |
|  |  | Pb | 6.56E-06 | 1.18E-05 | 1.99E-05 | 6.04E-05 | 2.82E-04 | 5.36E-04 |
|  |  | Hg | 4.31E-35 | 1.27E-09 | 6.94E-02 | 1.03E-01 | 1.92E-01 | 2.92E-01 |
|  |  | Mo | 3.04E-54 | 9.35E-17 | 1.25E-06 | 1.79E-06 | 3.24E-06 | 4.91E-06 |
|  |  | Ni | 7.32E-46 | 1.81E-12 | 3.15E-03 | 4.51E-03 | 8.26E-03 | 2.67E-02 |
|  |  | Se | 1.25E-09 | 2.85E-06 | 6.60E-06 | 8.91E-06 | 1.64E-05 | 5.11E-05 |
|  |  |  |  |  |  |  |  |  |
|  | Boys | Al | 1.54E-06 | 3.63E-06 | 5.62E-06 | 8.17E-06 | 1.52E-05 | 2.51E-05 |
|  |  | Ba | 3.21E-23 | 4.74E-09 | 8.49E-05 | 1.25E-04 | 2.32E-04 | 3.34E-04 |
|  |  | Cd | 4.66E-26 | 1.12E-08 | 1.59E-02 | 2.40E-02 | 4.62E-02 | 6.49E-02 |
|  |  | Cr | 4.19E-13 | 2.30E-06 | 1.27E-05 | 1.77E-05 | 3.22E-05 | 4.46E-05 |
|  |  | Cu | 4.27E-05 | 9.51E-05 | 1.29E-04 | 1.77E-04 | 3.25E-04 | 4.71E-04 |
|  |  | Pb | 3.07E-06 | 5.95E-06 | 9.15E-06 | 3.47E-05 | 1.63E-04 | 2.65E-04 |
|  |  | Hg | 1.86E-34 | 7.06E-10 | 3.61E-02 | 5.30E-02 | 9.86E-02 | 1.42E-01 |
|  |  | Mo | 5.20E-52 | 1.72E-16 | 6.15E-07 | 8.97E-07 | 1.70E-06 | 2.39E-06 |
|  |  | Ni | 5.34E-45 | 8.69E-13 | 1.56E-03 | 2.27E-03 | 4.33E-03 | 6.08E-03 |
|  |  | Se | 1.04E-09 | 1.59E-06 | 3.35E-06 | 4.64E-06 | 8.58E-06 | 1.17E-05 |
|  |  |  |  |  |  |  |  |  |
|  | Women | Al | 1.86E-06 | 4.29E-06 | 6.56E-06 | 9.14E-06 | 1.40E-05 | 2.35E-05 |
|  |  | Ba | 2.40E-23 | 4.57E-09 | 1.09E-04 | 1.40E-04 | 1.93E-04 | 3.41E-04 |
|  |  | Cd | 1.31E-25 | 2.42E-08 | 2.04E-02 | 2.75E-02 | 3.83E-02 | 7.24E-02 |
|  |  | Cr | 3.78E-13 | 2.04E-06 | 1.58E-05 | 1.94E-05 | 2.64E-05 | 5.03E-05 |
|  |  | Cu | 5.10E-05 | 1.16E-04 | 1.55E-04 | 1.91E-04 | 2.62E-04 | 5.11E-04 |
|  |  | Pb | 3.82E-06 | 7.08E-06 | 9.75E-06 | 3.90E-05 | 1.69E-04 | 2.27E-04 |
|  |  | Hg | 3.78E-33 | 6.20E-10 | 4.64E-02 | 6.00E-02 | 8.20E-02 | 1.45E-01 |
|  |  | Mo | 2.14E-51 | 1.94E-16 | 8.21E-07 | 1.02E-06 | 1.41E-06 | 2.43E-06 |
|  |  | Ni | 1.70E-45 | 4.56E-13 | 2.07E-03 | 2.59E-03 | 3.59E-03 | 6.20E-03 |
|  |  | Se | 1.83E-09 | 1.86E-06 | 4.16E-06 | 5.09E-06 | 6.94E-06 | 1.32E-05 |
|  |  |  |  |  |  |  |  |  |
|  | Men | Al | 6.69E-06 | 1.50E-05 | 2.31E-05 | 3.22E-05 | 4.57E-05 | 5.90E-05 |
|  |  | Ba | 6.70E-23 | 3.07E-08 | 3.99E-04 | 5.12E-04 | 6.65E-04 | 7.85E-04 |
|  |  | Cd | 5.68E-25 | 6.47E-08 | 7.38E-02 | 1.01E-01 | 1.31E-01 | 1.57E-01 |
|  |  | Cr | 1.31E-12 | 7.41E-06 | 5.69E-05 | 7.01E-05 | 8.96E-05 | 1.05E-04 |
|  |  | Cu | 1.90E-04 | 4.10E-04 | 5.52E-04 | 6.80E-04 | 8.87E-04 | 1.09E-03 |
|  |  | Pb | 1.43E-05 | 2.52E-05 | 3.81E-05 | 1.26E-04 | 5.70E-04 | 7.65E-04 |
|  |  | Hg | 1.40E-33 | 1.60E-09 | 1.63E-01 | 2.19E-01 | 2.83E-01 | 3.34E-01 |
|  |  | Mo | 4.66E-52 | 1.33E-15 | 2.96E-06 | 3.70E-06 | 4.80E-06 | 5.61E-06 |
|  |  | Ni | 4.42E-45 | 3.02E-12 | 7.36E-03 | 9.36E-03 | 1.22E-02 | 1.43E-02 |
|  |  | Se | 2.89E-09 | 5.96E-06 | 1.49E-05 | 1.82E-05 | 2.36E-05 | 2.76E-05 |
|  |  |  |  |  |  |  |  |  |
| Beaver | Girls | Al | 2.19E-06 | 4.28E-06 | 6.47E-06 | 9.63E-06 | 1.84E-05 | 4.80E-05 |
|  |  | Cd | 8.86E-42 | 1.85E-16 | 2.86E-06 | 4.30E-02 | 8.85E-02 | 1.30E-01 |
|  |  | Cr | 6.95E-12 | 9.48E-08 | 4.36E-06 | 2.44E-05 | 5.18E-05 | 8.35E-05 |
|  |  | Cu | 3.13E-05 | 1.61E-04 | 2.78E-04 | 3.91E-04 | 7.30E-04 | 2.05E-03 |
|  |  | Pb | 4.83E-06 | 9.85E-06 | 1.90E-05 | 3.62E-05 | 6.44E-04 | 9.62E-04 |
|  |  | Hg | 5.34E-70 | 9.48E-21 | 7.19E-02 | 1.23E-01 | 2.31E-01 | 3.55E-01 |
|  |  |  |  |  |  |  |  |  |
|  | Boys | Al | 1.69E-06 | 3.16E-06 | 4.72E-06 | 7.04E-06 | 1.27E-05 | 2.08E-05 |
|  |  | Cd | 3.99E-39 | 3.00E-16 | 1.66E-06 | 3.07E-02 | 6.14E-02 | 1.03E-01 |
|  |  | Cr | 8.43E-12 | 8.20E-08 | 3.44E-06 | 1.81E-05 | 3.73E-05 | 6.42E-05 |
|  |  | Cu | 2.47E-05 | 1.19E-04 | 1.99E-04 | 2.84E-04 | 5.33E-04 | 7.78E-04 |
|  |  | Pb | 3.31E-06 | 6.45E-06 | 1.28E-05 | 2.57E-05 | 4.48E-04 | 9.83E-04 |
|  |  | Hg | 7.08E-65 | 1.11E-20 | 5.64E-02 | 8.80E-02 | 1.70E-01 | 2.39E-01 |
|  |  |  |  |  |  |  |  |  |
|  | Women | Al | 4.93E-06 | 9.14E-06 | 1.34E-05 | 1.85E-05 | 2.94E-05 | 4.86E-05 |
|  |  | Cd | 1.41E-39 | 4.74E-16 | 1.12E-05 | 9.81E-02 | 1.47E-01 | 1.96E-01 |
|  |  | Cr | 2.88E-11 | 2.30E-07 | 9.56E-06 | 5.13E-05 | 8.74E-05 | 1.26E-04 |
|  |  | Cu | 7.30E-05 | 3.45E-04 | 5.90E-04 | 7.62E-04 | 1.10E-03 | 2.00E-03 |
|  |  | Pb | 1.02E-05 | 2.01E-05 | 3.96E-05 | 5.83E-05 | 1.21E-03 | 2.28E-03 |
|  |  | Hg | 3.30E-65 | 1.63E-19 | 1.79E-01 | 2.48E-01 | 3.36E-01 | 6.12E-01 |
|  |  |  |  |  |  |  |  |  |
|  | Men | Al | 1.09E-05 | 2.01E-05 | 2.88E-05 | 3.96E-05 | 5.92E-05 | 7.65E-05 |
|  |  | Cd | 1.58E-38 | 5.71E-16 | 5.74E-06 | 2.07E-01 | 3.01E-01 | 3.85E-01 |
|  |  | Cr | 6.00E-11 | 4.17E-07 | 2.12E-05 | 1.13E-04 | 1.83E-04 | 2.32E-04 |
|  |  | Cu | 1.43E-04 | 7.43E-04 | 1.28E-03 | 1.66E-03 | 2.18E-03 | 2.72E-03 |
|  |  | Pb | 2.26E-05 | 4.11E-05 | 8.84E-05 | 1.23E-04 | 2.18E-03 | 4.54E-03 |
|  |  | Hg | 2.47E-63 | 1.77E-18 | 3.90E-01 | 5.50E-01 | 7.13E-01 | 8.57E-01 |
|  |  |  |  |  |  |  |  |  |
| Caribou | Girls | Ba | 1.02E-17 | 6.10E-07 | 2.25E-04 | 3.19E-04 | 5.84E-04 | 1.32E-03 |
|  |  | Cd | 8.74E-40 | 4.45E-02 | 5.64E-02 | 7.19E-02 | 1.29E-01 | 4.07E-01 |
|  |  | Cu | 1.48E-04 | 2.49E-04 | 3.35E-04 | 4.44E-04 | 7.95E-04 | 2.35E-03 |
|  |  | Pb | 7.45E-06 | 1.08E-05 | 1.97E-05 | 2.85E-05 | 5.76E-05 | 1.23E-04 |
|  |  | Hg | 3.00E-89 | 4.00E-25 | 7.89E-02 | 1.30E-01 | 2.45E-01 | 3.76E-01 |
|  |  | Ni | 1.91E-39 | 4.43E-10 | 4.16E-03 | 5.87E-03 | 1.10E-02 | 3.44E-02 |
|  |  | Se | 2.25E-07 | 5.25E-06 | 8.62E-06 | 1.15E-05 | 2.08E-05 | 6.59E-05 |
|  |  |  |  |  |  |  |  |  |
|  | Boys | Ba | 6.17E-17 | 6.74E-07 | 2.66E-04 | 3.82E-04 | 7.25E-04 | 9.91E-04 |
|  |  | Cd | 7.15E-36 | 5.23E-02 | 6.71E-02 | 9.09E-02 | 1.72E-01 | 3.05E-01 |
|  |  | Cu | 1.90E-04 | 3.08E-04 | 3.97E-04 | 5.39E-04 | 9.85E-04 | 1.44E-03 |
|  |  | Pb | 8.67E-06 | 1.35E-05 | 2.56E-05 | 3.63E-05 | 5.91E-05 | 8.67E-05 |
|  |  | Hg | 1.05E-82 | 8.71E-23 | 1.05E-01 | 1.57E-01 | 3.00E-01 | 4.21E-01 |
|  |  | Ni | 9.49E-38 | 6.09E-10 | 4.98E-03 | 7.05E-03 | 1.32E-02 | 1.85E-02 |
|  |  | Se | 3.41E-07 | 6.93E-06 | 1.02E-05 | 1.40E-05 | 2.58E-05 | 3.57E-05 |
|  |  |  |  |  |  |  |  |  |
|  | Women | Ba | 5.64E-17 | 7.35E-07 | 3.02E-04 | 3.76E-04 | 5.14E-04 | 9.57E-04 |
|  |  | Cd | 6.93E-34 | 6.10E-02 | 6.98E-02 | 8.39E-02 | 1.14E-01 | 2.35E-01 |
|  |  | Cu | 1.96E-04 | 3.27E-04 | 4.12E-04 | 5.01E-04 | 6.98E-04 | 1.31E-03 |
|  |  | Pb | 1.03E-05 | 1.36E-05 | 2.45E-05 | 3.64E-05 | 4.82E-05 | 6.57E-05 |
|  |  | Hg | 6.47E-82 | 2.88E-23 | 1.19E-01 | 1.58E-01 | 2.12E-01 | 3.87E-01 |
|  |  | Ni | 1.15E-37 | 4.07E-10 | 5.61E-03 | 6.92E-03 | 9.56E-03 | 1.83E-02 |
|  |  | Se | 4.11E-07 | 7.39E-06 | 1.10E-05 | 1.34E-05 | 1.85E-05 | 3.49E-05 |
|  |  |  |  |  |  |  |  |  |
|  | Men | Ba | 8.93E-17 | 1.96E-06 | 8.64E-04 | 1.10E-03 | 1.42E-03 | 1.67E-03 |
|  |  | Cd | 5.11E-35 | 1.73E-01 | 2.01E-01 | 2.35E-01 | 2.96E-01 | 4.21E-01 |
|  |  | Cu | 5.59E-04 | 9.39E-04 | 1.18E-03 | 1.42E-03 | 1.84E-03 | 2.25E-03 |
|  |  | Pb | 2.77E-05 | 3.71E-05 | 7.38E-05 | 1.01E-04 | 1.42E-04 | 1.62E-04 |
|  |  | Hg | 3.20E-80 | 2.06E-22 | 3.37E-01 | 4.60E-01 | 6.03E-01 | 7.12E-01 |
|  |  | Ni | 1.64E-37 | 2.94E-09 | 1.63E-02 | 2.02E-02 | 2.60E-02 | 3.04E-02 |
|  |  | Se | 1.05E-06 | 2.06E-05 | 3.17E-05 | 3.85E-05 | 4.96E-05 | 5.87E-05 |
|  |  |  |  |  |  |  |  |  |
| Duck | Girls | Al | 1.16E-06 | 6.60E-06 | 1.17E-05 | 1.71E-05 | 3.19E-05 | 9.25E-05 |
|  |  | Ba | 5.08E-14 | 9.92E-08 | 3.33E-05 | 1.53E-04 | 2.96E-04 | 4.21E-04 |
|  |  | Cd | 3.06E-47 | 4.00E-15 | 2.04E-02 | 3.72E-02 | 7.04E-02 | 1.08E-01 |
|  |  | Cr | 8.87E-09 | 1.53E-06 | 9.60E-06 | 1.98E-05 | 3.75E-05 | 6.54E-05 |
|  |  | Cu | 7.33E-05 | 1.22E-04 | 1.66E-04 | 2.27E-04 | 4.08E-04 | 1.19E-03 |
|  |  | Pb | 4.58E-06 | 8.80E-06 | 1.75E-05 | 2.74E-05 | 5.51E-05 | 1.18E-04 |
|  |  | Hg | 3.04E-04 | 9.53E-03 | 3.44E-02 | 6.26E-02 | 1.19E-01 | 2.28E-01 |
|  |  | Mo | 5.53E-43 | 6.50E-10 | 1.11E-06 | 1.45E-06 | 2.65E-06 | 8.25E-06 |
|  |  | Se | 6.18E-07 | 2.10E-06 | 3.56E-06 | 5.36E-06 | 1.00E-05 | 2.62E-05 |
|  |  |  |  |  |  |  |  |  |
|  | Boys | Al | 3.59E-06 | 1.77E-05 | 3.12E-05 | 4.55E-05 | 8.39E-05 | 1.25E-04 |
|  |  | Ba | 7.45E-13 | 3.51E-07 | 9.16E-05 | 4.00E-04 | 7.52E-04 | 1.23E-03 |
|  |  | Cd | 1.67E-45 | 1.22E-14 | 5.93E-02 | 9.59E-02 | 1.84E-01 | 2.66E-01 |
|  |  | Cr | 2.79E-08 | 3.65E-06 | 2.49E-05 | 5.16E-05 | 9.75E-05 | 1.59E-04 |
|  |  | Cu | 1.97E-04 | 3.30E-04 | 4.39E-04 | 6.05E-04 | 1.10E-03 | 1.65E-03 |
|  |  | Pb | 1.22E-05 | 2.55E-05 | 4.41E-05 | 7.00E-05 | 1.19E-04 | 2.06E-04 |
|  |  | Hg | 1.06E-03 | 2.67E-02 | 9.41E-02 | 1.64E-01 | 3.12E-01 | 5.03E-01 |
|  |  | Mo | 1.39E-42 | 4.61E-10 | 2.83E-06 | 3.90E-06 | 7.20E-06 | 9.77E-06 |
|  |  | Se | 1.76E-06 | 5.56E-06 | 9.43E-06 | 1.40E-05 | 2.53E-05 | 4.01E-05 |
|  |  |  |  |  |  |  |  |  |
|  | Women | Al | 6.02E-06 | 3.21E-05 | 5.82E-05 | 7.84E-05 | 1.12E-04 | 2.05E-04 |
|  |  | Ba | 4.04E-13 | 4.72E-07 | 1.38E-04 | 7.58E-04 | 1.11E-03 | 1.50E-03 |
|  |  | Cd | 1.23E-45 | 8.88E-14 | 1.17E-01 | 1.73E-01 | 2.37E-01 | 4.36E-01 |
|  |  | Cr | 5.49E-08 | 5.87E-06 | 4.28E-05 | 9.40E-05 | 1.42E-04 | 1.98E-04 |
|  |  | Cu | 3.83E-04 | 6.21E-04 | 7.99E-04 | 1.01E-03 | 1.43E-03 | 2.71E-03 |
|  |  | Pb | 2.57E-05 | 4.14E-05 | 8.01E-05 | 1.21E-04 | 1.72E-04 | 3.01E-04 |
|  |  | Hg | 1.72E-03 | 4.83E-02 | 1.70E-01 | 2.97E-01 | 4.48E-01 | 6.89E-01 |
|  |  | Mo | 1.55E-41 | 1.98E-09 | 5.55E-06 | 6.78E-06 | 9.26E-06 | 1.72E-05 |
|  |  | Se | 3.24E-06 | 1.03E-05 | 1.74E-05 | 2.41E-05 | 3.53E-05 | 6.25E-05 |
|  |  |  |  |  |  |  |  |  |
|  | Men | Al | 9.29E-06 | 4.80E-05 | 8.67E-05 | 1.17E-04 | 1.56E-04 | 1.95E-04 |
|  |  | Ba | 6.87E-13 | 7.60E-07 | 2.42E-04 | 1.15E-03 | 1.56E-03 | 1.98E-03 |
|  |  | Cd | 9.16E-43 | 1.08E-13 | 1.84E-01 | 2.64E-01 | 3.44E-01 | 4.16E-01 |
|  |  | Cr | 5.68E-08 | 8.96E-06 | 6.50E-05 | 1.40E-04 | 1.98E-04 | 2.49E-04 |
|  |  | Cu | 5.81E-04 | 9.23E-04 | 1.19E-03 | 1.48E-03 | 1.97E-03 | 2.47E-03 |
|  |  | Pb | 3.93E-05 | 6.28E-05 | 1.19E-04 | 1.73E-04 | 2.45E-04 | 3.23E-04 |
|  |  | Hg | 2.01E-03 | 6.68E-02 | 2.43E-01 | 4.32E-01 | 6.24E-01 | 8.02E-01 |
|  |  | Mo | 8.30E-42 | 8.09E-09 | 8.40E-06 | 1.01E-05 | 1.29E-05 | 1.49E-05 |
|  |  | Se | 5.05E-06 | 1.53E-05 | 2.55E-05 | 3.52E-05 | 4.92E-05 | 6.15E-05 |
|  |  |  |  |  |  |  |  |  |
| Goose | Girls | Sb | 1.33E-148 | 1.06E-61 | 1.94E-24 | 3.89E-04 | 8.13E-04 | 1.61E-03 |
|  |  | Ba | 5.70E-43 | 1.36E-15 | 1.44E-04 | 1.12E-03 | 2.23E-03 | 3.47E-03 |
|  |  | Cu | 4.11E-04 | 7.46E-04 | 1.05E-03 | 1.43E-03 | 2.58E-03 | 7.22E-03 |
|  |  | Pb | 2.40E-05 | 6.53E-05 | 1.04E-04 | 2.69E-04 | 1.72E-03 | 1.95E-02 |
|  |  | Mo | 1.55E-43 | 1.08E-08 | 7.12E-06 | 9.39E-06 | 1.71E-05 | 5.30E-05 |
|  |  | Se | 6.85E-10 | 6.94E-06 | 3.15E-05 | 4.44E-05 | 8.09E-05 | 2.60E-04 |
|  |  |  |  |  |  |  |  |  |
|  | Boys | Sb | 2.73E-148 | 1.23E-61 | 2.96E-24 | 5.41E-04 | 1.15E-03 | 2.21E-03 |
|  |  | Ba | 1.20E-41 | 9.44E-16 | 1.99E-04 | 1.57E-03 | 2.95E-03 | 4.33E-03 |
|  |  | Cu | 6.13E-04 | 1.09E-03 | 1.48E-03 | 2.05E-03 | 3.69E-03 | 5.73E-03 |
|  |  | Pb | 4.41E-05 | 9.67E-05 | 1.42E-04 | 3.69E-04 | 2.73E-03 | 4.42E-02 |
|  |  | Mo | 1.25E-41 | 5.85E-08 | 9.86E-06 | 1.37E-05 | 2.50E-05 | 3.39E-05 |
|  |  | Se | 8.49E-10 | 1.03E-05 | 4.56E-05 | 6.34E-05 | 1.20E-04 | 1.66E-04 |
|  |  |  |  |  |  |  |  |  |
|  | Women | Sb | 2.64E-148 | 1.74E-61 | 3.11E-24 | 7.62E-04 | 1.30E-03 | 1.66E-03 |
|  |  | Ba | 2.18E-40 | 4.39E-15 | 2.34E-04 | 1.97E-03 | 2.77E-03 | 5.11E-03 |
|  |  | Cu | 8.02E-04 | 1.40E-03 | 1.84E-03 | 2.35E-03 | 3.36E-03 | 6.25E-03 |
|  |  | Pb | 4.31E-05 | 1.27E-04 | 1.65E-04 | 5.35E-04 | 2.25E-03 | 4.38E-02 |
|  |  | Mo | 4.79E-41 | 9.73E-08 | 1.32E-05 | 1.60E-05 | 2.17E-05 | 4.04E-05 |
|  |  | Se | 1.42E-09 | 1.22E-05 | 6.09E-05 | 7.49E-05 | 1.04E-04 | 1.92E-04 |
|  |  |  |  |  |  |  |  |  |
|  | Men | Sb | 3.04E-148 | 2.86E-61 | 5.43E-24 | 1.20E-03 | 1.88E-03 | 2.38E-03 |
|  |  | Ba | 6.25E-39 | 4.80E-15 | 5.06E-04 | 3.03E-03 | 3.99E-03 | 4.93E-03 |
|  |  | Cu | 1.19E-03 | 2.09E-03 | 2.79E-03 | 3.50E-03 | 4.67E-03 | 5.83E-03 |
|  |  | Pb | 6.70E-05 | 2.01E-04 | 2.45E-04 | 6.77E-04 | 3.46E-03 | 6.40E-02 |
|  |  | Mo | 2.21E-42 | 2.59E-08 | 2.01E-05 | 2.42E-05 | 3.09E-05 | 3.56E-05 |
|  |  | Se | 1.42E-09 | 1.93E-05 | 9.24E-05 | 1.15E-04 | 1.48E-04 | 1.75E-04 |
|  |  |  |  |  |  |  |  |  |
| Moose | Girls | Al | 5.75E-06 | 8.34E-06 | 1.10E-05 | 1.51E-05 | 2.64E-05 | 8.19E-05 |
|  |  | Ba | 4.65E-09 | 4.08E-06 | 6.64E-05 | 3.02E-04 | 7.68E-04 | 1.46E-03 |
|  |  | Cd | 8.29E-10 | 3.28E-05 | 2.41E-03 | 3.07E-02 | 1.28E-01 | 2.42E-01 |
|  |  | Cr | 3.77E-08 | 2.58E-06 | 1.64E-05 | 4.57E-05 | 1.04E-04 | 1.97E-04 |
|  |  | Cu | 7.48E-05 | 1.15E-04 | 1.55E-04 | 2.14E-04 | 3.87E-04 | 1.08E-03 |
|  |  | Pb | 1.35E-05 | 4.14E-05 | 5.82E-05 | 1.30E-04 | 7.91E-04 | 8.59E-03 |
|  |  | Se | 2.99E-16 | 5.01E-09 | 3.65E-06 | 2.04E-05 | 4.04E-05 | 6.90E-05 |
|  |  |  |  |  |  |  |  |  |
|  | Boys | Al | 7.12E-06 | 9.90E-06 | 1.30E-05 | 1.78E-05 | 3.24E-05 | 5.14E-05 |
|  |  | Ba | 1.20E-08 | 5.82E-06 | 8.07E-05 | 3.55E-04 | 8.86E-04 | 1.64E-03 |
|  |  | Cd | 1.44E-09 | 3.92E-05 | 2.92E-03 | 3.38E-02 | 1.39E-01 | 2.51E-01 |
|  |  | Cr | 4.45E-08 | 3.34E-06 | 1.92E-05 | 5.47E-05 | 1.18E-04 | 2.04E-04 |
|  |  | Cu | 9.56E-05 | 1.38E-04 | 1.83E-04 | 2.50E-04 | 4.52E-04 | 7.39E-04 |
|  |  | Pb | 1.81E-05 | 4.62E-05 | 6.72E-05 | 1.46E-04 | 1.29E-03 | 8.27E-03 |
|  |  | Se | 1.76E-15 | 5.46E-09 | 3.88E-06 | 2.32E-05 | 4.33E-05 | 7.19E-05 |
|  |  |  |  |  |  |  |  |  |
|  | Women | Al | 1.83E-05 | 2.52E-05 | 3.09E-05 | 3.86E-05 | 5.63E-05 | 1.04E-04 |
|  |  | Ba | 2.73E-08 | 1.27E-05 | 1.97E-04 | 8.69E-04 | 1.90E-03 | 2.75E-03 |
|  |  | Cd | 5.10E-09 | 1.05E-04 | 6.60E-03 | 8.00E-02 | 3.13E-01 | 4.74E-01 |
|  |  | Cr | 1.28E-07 | 8.13E-06 | 4.62E-05 | 1.27E-04 | 2.50E-04 | 3.80E-04 |
|  |  | Cu | 2.43E-04 | 3.45E-04 | 4.36E-04 | 5.55E-04 | 8.23E-04 | 1.49E-03 |
|  |  | Pb | 3.65E-05 | 1.20E-04 | 1.61E-04 | 3.76E-04 | 2.57E-03 | 3.01E-02 |
|  |  | Se | 5.36E-15 | 1.99E-08 | 9.43E-06 | 5.92E-05 | 8.70E-05 | 1.16E-04 |
|  |  |  |  |  |  |  |  |  |
|  | Men | Al | 2.68E-05 | 3.61E-05 | 4.43E-05 | 5.44E-05 | 7.32E-05 | 9.13E-05 |
|  |  | Ba | 4.45E-08 | 1.92E-05 | 2.64E-04 | 1.16E-03 | 2.62E-03 | 3.41E-03 |
|  |  | Cd | 3.75E-09 | 1.08E-04 | 8.76E-03 | 1.10E-01 | 4.51E-01 | 6.24E-01 |
|  |  | Cr | 1.69E-07 | 1.11E-05 | 6.47E-05 | 1.81E-04 | 3.46E-04 | 4.56E-04 |
|  |  | Cu | 3.53E-04 | 4.96E-04 | 6.24E-04 | 7.82E-04 | 1.08E-03 | 1.43E-03 |
|  |  | Pb | 5.54E-05 | 1.82E-04 | 2.24E-04 | 5.13E-04 | 3.93E-03 | 3.51E-02 |
|  |  | Se | 2.65E-15 | 2.14E-08 | 1.35E-05 | 8.51E-05 | 1.19E-04 | 1.50E-04 |
|  |  |  |  |  |  |  |  |  |
| Grouse | Girls | Ba | 1.66E-15 | 2.86E-08 | 2.66E-05 | 2.00E-04 | 4.04E-04 | 6.20E-04 |
|  |  | Cd | 6.23E-72 | 9.91E-25 | 4.71E-06 | 4.53E-02 | 8.86E-02 | 1.32E-01 |
|  |  | Cu | 5.55E-05 | 1.46E-04 | 2.18E-04 | 3.11E-04 | 5.79E-04 | 1.72E-03 |
|  |  | Pb | 2.41E-06 | 1.58E-05 | 2.58E-05 | 3.58E-04 | 6.67E-03 | 8.61E-03 |
|  |  | Mo | 5.34E-49 | 2.09E-13 | 1.35E-06 | 1.90E-06 | 3.52E-06 | 1.10E-05 |
|  |  | Se | 6.61E-09 | 2.50E-06 | 6.56E-06 | 9.18E-06 | 1.67E-05 | 5.34E-05 |
|  |  | Tin | 1.34E-30 | 1.03E-08 | 2.00E-05 | 2.69E-05 | 4.94E-05 | 1.54E-04 |
|  |  |  |  |  |  |  |  |  |
|  | Boys | Ba | 9.26E-15 | 6.36E-08 | 4.28E-05 | 2.48E-04 | 4.63E-04 | 7.73E-04 |
|  |  | Cd | 6.14E-67 | 3.77E-24 | 2.29E-05 | 5.59E-02 | 1.01E-01 | 1.58E-01 |
|  |  | Cu | 7.62E-05 | 1.88E-04 | 2.78E-04 | 3.94E-04 | 7.10E-04 | 1.09E-03 |
|  |  | Pb | 3.69E-06 | 1.89E-05 | 3.19E-05 | 5.02E-04 | 6.77E-03 | 1.06E-02 |
|  |  | Mo | 3.69E-45 | 1.41E-12 | 1.75E-06 | 2.40E-06 | 4.56E-06 | 6.19E-06 |
|  |  | Se | 1.33E-08 | 3.03E-06 | 8.17E-06 | 1.15E-05 | 2.12E-05 | 3.04E-05 |
|  |  | Tin | 3.75E-29 | 1.98E-08 | 2.47E-05 | 3.44E-05 | 6.39E-05 | 8.66E-05 |
|  |  |  |  |  |  |  |  |  |
|  | Women | Ba | 1.36E-14 | 5.46E-08 | 4.71E-05 | 4.01E-04 | 6.02E-04 | 8.73E-04 |
|  |  | Cd | 6.22E-66 | 1.08E-23 | 4.01E-05 | 8.87E-02 | 1.25E-01 | 1.98E-01 |
|  |  | Cu | 1.25E-04 | 2.93E-04 | 4.36E-04 | 5.67E-04 | 8.24E-04 | 1.58E-03 |
|  |  | Pb | 5.52E-06 | 3.15E-05 | 5.12E-05 | 7.73E-04 | 1.02E-02 | 1.36E-02 |
|  |  | Mo | 5.72E-46 | 2.73E-13 | 2.91E-06 | 3.53E-06 | 4.87E-06 | 9.29E-06 |
|  |  | Se | 1.44E-08 | 5.22E-06 | 1.38E-05 | 1.71E-05 | 2.35E-05 | 4.51E-05 |
|  |  | Tin | 9.52E-30 | 5.07E-08 | 4.15E-05 | 5.04E-05 | 6.99E-05 | 1.30E-04 |
|  |  |  |  |  |  |  |  |  |
|  | Men | Ba | 3.44E-14 | 2.25E-07 | 1.47E-04 | 1.03E-03 | 1.43E-03 | 1.82E-03 |
|  |  | Cd | 5.94E-66 | 3.22E-23 | 5.36E-05 | 2.25E-01 | 3.02E-01 | 3.78E-01 |
|  |  | Cu | 3.00E-04 | 7.43E-04 | 1.10E-03 | 1.43E-03 | 1.92E-03 | 2.42E-03 |
|  |  | Pb | 1.59E-05 | 7.69E-05 | 1.22E-04 | 2.21E-03 | 2.90E-02 | 4.15E-02 |
|  |  | Mo | 5.59E-46 | 1.64E-12 | 7.38E-06 | 9.19E-06 | 1.17E-05 | 1.37E-05 |
|  |  | Se | 3.79E-08 | 1.29E-05 | 3.52E-05 | 4.36E-05 | 5.62E-05 | 6.71E-05 |
|  |  | Tin | 2.06E-29 | 2.68E-08 | 1.05E-04 | 1.29E-04 | 1.64E-04 | 1.91E-04 |
|  |  |  |  |  |  |  |  |  |
| Hare | Girls | Ba | 1.60E-07 | 7.26E-06 | 3.82E-05 | 1.12E-04 | 2.73E-04 | 5.13E-04 |
|  |  | Cd | 1.42E-92 | 3.57E-34 | 1.11E-09 | 5.03E-02 | 9.90E-02 | 1.59E-01 |
|  |  | Cr | 4.93E-13 | 3.71E-08 | 4.46E-06 | 2.82E-05 | 5.85E-05 | 1.06E-04 |
|  |  | Cu | 1.03E-04 | 2.20E-04 | 3.08E-04 | 4.20E-04 | 7.58E-04 | 2.36E-03 |
|  |  | Se | 1.27E-13 | 1.20E-07 | 6.92E-06 | 1.05E-05 | 1.98E-05 | 3.90E-05 |
|  |  |  |  |  |  |  |  |  |
|  | Boys | Ba | 3.23E-07 | 1.26E-05 | 6.42E-05 | 1.84E-04 | 4.50E-04 | 7.74E-04 |
|  |  | Cd | 9.26E-87 | 1.06E-32 | 5.59E-09 | 8.26E-02 | 1.54E-01 | 2.49E-01 |
|  |  | Cr | 4.88E-13 | 5.44E-08 | 6.49E-06 | 4.51E-05 | 9.15E-05 | 1.56E-04 |
|  |  | Cu | 1.85E-04 | 3.72E-04 | 5.07E-04 | 6.98E-04 | 1.29E-03 | 1.89E-03 |
|  |  | Se | 4.07E-13 | 2.39E-07 | 1.18E-05 | 1.74E-05 | 3.36E-05 | 4.71E-05 |
|  |  |  |  |  |  |  |  |  |
|  | Women | Ba | 4.54E-07 | 2.02E-05 | 1.07E-04 | 2.94E-04 | 6.38E-04 | 9.44E-04 |
|  |  | Cd | 9.88E-88 | 3.89E-33 | 3.84E-09 | 1.42E-01 | 2.03E-01 | 2.68E-01 |
|  |  | Cr | 1.31E-12 | 1.14E-07 | 1.14E-05 | 7.72E-05 | 1.26E-04 | 1.71E-04 |
|  |  | Cu | 2.98E-04 | 6.18E-04 | 8.33E-04 | 1.04E-03 | 1.45E-03 | 2.74E-03 |
|  |  | Se | 8.50E-13 | 4.05E-07 | 2.09E-05 | 2.74E-05 | 3.73E-05 | 6.84E-05 |
|  |  |  |  |  |  |  |  |  |
|  | Men | Ba | 1.11E-06 | 4.06E-05 | 2.17E-04 | 6.08E-04 | 1.29E-03 | 1.79E-03 |
|  |  | Cd | 9.09E-85 | 3.82E-31 | 4.57E-08 | 3.06E-01 | 4.19E-01 | 5.27E-01 |
|  |  | Cr | 3.63E-12 | 1.99E-07 | 2.28E-05 | 1.64E-04 | 2.53E-04 | 3.25E-04 |
|  |  | Cu | 6.57E-04 | 1.32E-03 | 1.78E-03 | 2.19E-03 | 2.90E-03 | 3.55E-03 |
|  |  | Se | 1.51E-12 | 6.89E-07 | 4.33E-05 | 5.89E-05 | 7.69E-05 | 9.25E-05 |
|  |  |  |  |  |  |  |  |  |
| Walleye | Girls | Ba | 2.23E-68 | 1.46E-25 | 6.64E-06 | 1.67E-04 | 3.30E-04 | 5.07E-04 |
|  |  | Cu | 2.29E-06 | 1.45E-04 | 2.10E-04 | 2.74E-04 | 4.94E-04 | 1.54E-03 |
|  |  | Pb | 1.14E-06 | 2.11E-06 | 3.38E-06 | 6.73E-06 | 1.93E-05 | 2.52E-05 |
|  |  | Hg | 3.74E-03 | 3.48E-02 | 5.59E-02 | 7.67E-02 | 1.41E-01 | 4.49E-01 |
|  |  | Se | 4.39E-07 | 4.16E-06 | 5.54E-06 | 7.15E-06 | 1.30E-05 | 3.81E-05 |
|  |  | Tin | 6.08E-25 | 9.75E-06 | 1.51E-05 | 1.93E-05 | 3.51E-05 | 1.09E-04 |
|  |  |  |  |  |  |  |  |  |
|  | Boys | Ba | 4.11E-68 | 5.74E-24 | 4.34E-05 | 7.59E-04 | 1.40E-03 | 2.14E-03 |
|  |  | Cu | 1.47E-05 | 6.93E-04 | 9.28E-04 | 1.29E-03 | 2.30E-03 | 3.13E-03 |
|  |  | Pb | 5.60E-06 | 1.07E-05 | 1.52E-05 | 3.06E-05 | 7.04E-05 | 1.10E-04 |
|  |  | Hg | 2.13E-02 | 1.60E-01 | 2.55E-01 | 3.52E-01 | 6.50E-01 | 9.33E-01 |
|  |  | Se | 2.77E-06 | 1.93E-05 | 2.49E-05 | 3.36E-05 | 6.38E-05 | 1.15E-04 |
|  |  | Tin | 2.41E-22 | 5.06E-05 | 6.75E-05 | 9.34E-05 | 1.71E-04 | 2.20E-04 |
|  |  |  |  |  |  |  |  |  |
|  | Women | Ba | 3.00E-65 | 2.06E-23 | 3.94E-05 | 1.06E-03 | 1.50E-03 | 2.76E-03 |
|  |  | Cu | 1.91E-05 | 1.06E-03 | 1.41E-03 | 1.71E-03 | 2.33E-03 | 4.34E-03 |
|  |  | Pb | 9.69E-06 | 1.39E-05 | 2.27E-05 | 3.42E-05 | 9.06E-05 | 1.63E-04 |
|  |  | Hg | 2.64E-02 | 2.26E-01 | 3.80E-01 | 4.78E-01 | 6.56E-01 | 1.27E+00 |
|  |  | Se | 3.86E-06 | 3.03E-05 | 3.59E-05 | 4.41E-05 | 6.05E-05 | 1.07E-04 |
|  |  | Tin | 2.73E-22 | 7.81E-05 | 1.02E-04 | 1.23E-04 | 1.68E-04 | 3.06E-04 |
|  |  |  |  |  |  |  |  |  |
|  | Men | Ba | 4.81E-66 | 6.33E-24 | 7.50E-05 | 1.60E-03 | 2.15E-03 | 2.61E-03 |
|  |  | Cu | 2.88E-05 | 1.58E-03 | 2.09E-03 | 2.50E-03 | 3.21E-03 | 3.71E-03 |
|  |  | Pb | 1.33E-05 | 2.08E-05 | 3.29E-05 | 4.55E-05 | 1.20E-04 | 1.56E-04 |
|  |  | Hg | 3.84E-02 | 3.31E-01 | 5.61E-01 | 7.01E-01 | 9.12E-01 | 1.10E+00 |
|  |  | Se | 4.77E-06 | 4.37E-05 | 5.34E-05 | 6.29E-05 | 8.03E-05 | 9.17E-05 |
|  |  | Tin | 3.20E-22 | 1.15E-04 | 1.51E-04 | 1.79E-04 | 2.29E-04 | 2.61E-04 |

*Key*:

P*_n_*: *n^t^*^h^-percentile
