# Supplementary material for: A probabilistic hazard and risk assessment of exposure to metals and organohalogens associated with a traditional diet in the Indigenous communities of Eeyou Istchee (northern Quebec, Canada)
Source: Environ Sci Pollut Res Int. 2022 Sep 24;30(6):14304–17. doi: 10.1007/s11356-022-23117-2 (PMC9908690; doi:10.1007/s11356-022-23117-2)
Supplement: Supplementary file 6 — (DOCX 56 kb) [file 11356_2022_23117_MOESM6_ESM.docx]

**Table S6: Probabilistic risk and hazard* for organohalogens for traditional foods at n > 5 and minimum level of detection > 50% of n of contaminant**

| **Species** | **Demographic** | **Contaminant** | **P_5_** | **P_25_** | **P_50_** | **P_75_** | **P_95_** | **P_99_** |
| --- | --- | --- | --- | --- | --- | --- | --- | --- |
| Bear | Girls | PCB 153 | 6.96E-12 | 1.28E-09 | 1.14E-08 | 5.02E-08 | 2.18E-07 | 5.21E-07 |
|  |  | PCB 170 | 8.55E-15 | 2.06E-10 | 1.22E-08 | 6.51E-08 | 1.37E-07 | 2.07E-07 |
|  |  | PCB 180 | 3.93E-11 | 4.33E-09 | 2.73E-08 | 6.44E-08 | 1.36E-07 | 2.47E-07 |
|  |  | PCB 52 | 1.16E-15 | 2.22E-09 | 6.86E-08 | 9.71E-08 | 1.81E-07 | 5.80E-07 |
|  |  |  |  |  |  |  |  |  |
|  | Boys | PCB 153 | 5.07E-12 | 7.52E-10 | 6.17E-09 | 2.65E-08 | 1.10E-07 | 2.30E-07 |
|  |  | PCB 170 | 1.26E-14 | 1.29E-10 | 6.55E-09 | 3.27E-08 | 6.72E-08 | 1.15E-07 |
|  |  | PCB 180 | 2.47E-11 | 2.37E-09 | 1.42E-08 | 3.30E-08 | 6.50E-08 | 1.12E-07 |
|  |  | PCB 52 | 1.50E-15 | 1.25E-09 | 3.43E-08 | 4.99E-08 | 9.27E-08 | 1.30E-07 |
|  |  |  |  |  |  |  |  |  |
|  | Women | PCB 153 | 5.90E-12 | 8.11E-10 | 6.80E-09 | 2.86E-08 | 1.14E-07 | 2.34E-07 |
|  |  | PCB 170 | 9.69E-15 | 1.27E-10 | 7.27E-09 | 3.98E-08 | 6.58E-08 | 9.16E-08 |
|  |  | PCB 180 | 2.97E-11 | 2.57E-09 | 1.59E-08 | 3.85E-08 | 6.16E-08 | 8.86E-08 |
|  |  | PCB 52 | 1.52E-15 | 1.42E-09 | 4.46E-08 | 5.58E-08 | 7.71E-08 | 1.51E-07 |
|  |  |  |  |  |  |  |  |  |
|  | Men | PCB 153 | 2.20E-11 | 3.02E-09 | 2.46E-08 | 1.05E-07 | 4.10E-07 | 7.58E-07 |
|  |  | PCB 170 | 4.67E-14 | 5.32E-10 | 2.63E-08 | 1.42E-07 | 2.21E-07 | 2.80E-07 |
|  |  | PCB 180 | 1.00E-10 | 9.59E-09 | 5.74E-08 | 1.37E-07 | 2.12E-07 | 2.65E-07 |
|  |  | PCB 52 | 6.71E-15 | 5.13E-09 | 1.59E-07 | 2.03E-07 | 2.61E-07 | 3.14E-07 |
|  |  |  |  |  |  |  |  |  |
| Duck | Girls | Cis-nonachlor | 1.67E-09 | 2.88E-09 | 4.03E-09 | 5.67E-09 | 1.03E-08 | 2.94E-08 |
|  |  | Mirex* | 4.59E-05 | 6.37E-05 | 8.05E-05 | 1.07E-04 | 1.85E-04 | 5.54E-04 |
|  |  | Oxychlordane | 3.14E-09 | 4.43E-09 | 5.71E-09 | 7.63E-09 | 1.33E-08 | 3.86E-08 |
|  |  | PBB 153 | 4.48E-08 | 1.03E-07 | 1.57E-07 | 2.32E-07 | 4.29E-07 | 1.14E-06 |
|  |  | PBDE 100* | 8.40E-05 | 1.18E-04 | 1.51E-04 | 2.03E-04 | 3.51E-04 | 1.03E-03 |
|  |  | PBDE 153* | 9.74E-05 | 1.37E-04 | 1.74E-04 | 2.34E-04 | 4.07E-04 | 1.20E-03 |
|  |  | PBDE 154* | 5.62E-05 | 1.02E-04 | 1.47E-04 | 2.10E-04 | 3.91E-04 | 1.12E-03 |
|  |  | PBDE 47* | 5.70E-05 | 8.30E-05 | 1.08E-04 | 1.48E-04 | 2.57E-04 | 7.68E-04 |
|  |  | PCB 101 | 1.14E-08 | 1.75E-08 | 2.32E-08 | 3.19E-08 | 5.83E-08 | 1.64E-07 |
|  |  | PCB 118 | 2.10E-08 | 2.81E-08 | 3.49E-08 | 4.64E-08 | 7.67E-08 | 2.35E-07 |
|  |  | PCB 128 | 1.95E-08 | 2.64E-08 | 3.29E-08 | 4.40E-08 | 7.33E-08 | 2.23E-07 |
|  |  | PCB 138 | 1.98E-08 | 2.65E-08 | 3.29E-08 | 4.36E-08 | 7.22E-08 | 2.19E-07 |
|  |  | PCB 153 | 3.99E-08 | 5.33E-08 | 6.62E-08 | 8.73E-08 | 1.44E-07 | 4.44E-07 |
|  |  | PCB 156 | 2.00E-08 | 2.76E-08 | 3.48E-08 | 4.63E-08 | 7.92E-08 | 2.33E-07 |
|  |  | PCB 163 | 1.78E-08 | 2.40E-08 | 3.00E-08 | 3.98E-08 | 6.75E-08 | 2.04E-07 |
|  |  | PCB 170 | 2.03E-08 | 2.74E-08 | 3.40E-08 | 4.52E-08 | 7.52E-08 | 2.28E-07 |
|  |  | PCB 180 | 2.06E-08 | 2.75E-08 | 3.43E-08 | 4.54E-08 | 7.53E-08 | 2.29E-07 |
|  |  | PCB 183 | 2.03E-08 | 2.74E-08 | 3.42E-08 | 4.55E-08 | 7.58E-08 | 2.34E-07 |
|  |  | PCB 187 | 2.08E-08 | 2.78E-08 | 3.47E-08 | 4.58E-08 | 7.56E-08 | 2.33E-07 |
|  |  | PCB 194 | 1.10E-08 | 2.32E-08 | 3.54E-08 | 5.09E-08 | 9.50E-08 | 2.53E-07 |
|  |  | PCB 28 | 1.99E-08 | 2.70E-08 | 3.38E-08 | 4.50E-08 | 7.60E-08 | 2.31E-07 |
|  |  | PCB 52 | 1.63E-08 | 2.22E-08 | 2.77E-08 | 3.70E-08 | 6.29E-08 | 1.88E-07 |
|  |  | PCB 99 | 1.61E-08 | 2.19E-08 | 2.72E-08 | 3.63E-08 | 6.08E-08 | 1.86E-07 |
|  |  | p-p’-DDD | 1.18E-09 | 2.04E-09 | 2.89E-09 | 4.12E-09 | 7.43E-09 | 2.05E-08 |
|  |  | p-p’-DDE | 3.61E-09 | 4.79E-09 | 5.99E-09 | 7.90E-09 | 1.30E-08 | 4.01E-08 |
|  |  | p-p’-DDT | 1.77E-09 | 2.78E-09 | 3.77E-09 | 5.25E-09 | 9.50E-09 | 2.51E-08 |
|  |  | β-Hexachlorocyclohexane | 3.33E-09 | 1.46E-08 | 2.82E-08 | 4.48E-08 | 8.69E-08 | 2.02E-07 |
|  |  | Toxaphene Parlar 26 | 3.54E-09 | 1.15E-08 | 2.00E-08 | 3.04E-08 | 5.85E-08 | 1.46E-07 |
|  |  | Toxaphene Parlar 32 | 7.65E-15 | 3.80E-10 | 1.96E-08 | 3.76E-08 | 7.36E-08 | 1.19E-07 |
|  |  | Toxaphene Parlar 50 | 3.71E-09 | 1.18E-08 | 2.01E-08 | 3.01E-08 | 5.68E-08 | 1.38E-07 |
|  |  | Trans-nonachlor | 1.82E-09 | 3.21E-09 | 4.53E-09 | 6.47E-09 | 1.18E-08 | 3.38E-08 |
|  |  |  |  |  |  |  |  |  |
|  | Boys | Cis-nonachlor | 4.56E-09 | 7.61E-09 | 1.07E-08 | 1.50E-08 | 2.71E-08 | 4.35E-08 |
|  |  | Mirex* | 1.30E-04 | 1.68E-04 | 2.12E-04 | 2.87E-04 | 5.05E-04 | 8.29E-04 |
|  |  | Oxychlordane | 8.76E-09 | 1.17E-08 | 1.50E-08 | 2.03E-08 | 3.61E-08 | 5.75E-08 |
|  |  | PBB 153 | 1.24E-07 | 2.79E-07 | 4.19E-07 | 6.09E-07 | **1.09E-06** | 1.78E-06 |
|  |  | PBDE 100* | 2.37E-04 | 3.12E-04 | 3.97E-04 | 5.38E-04 | 9.59E-04 | 1.53E-03 |
|  |  | PBDE 153* | 2.73E-04 | 3.63E-04 | 4.60E-04 | 6.20E-04 | 1.11E-03 | 1.81E-03 |
|  |  | PBDE 154* | 1.57E-04 | 2.75E-04 | 3.93E-04 | 5.58E-04 | 1.02E-03 | 1.65E-03 |
|  |  | PBDE 47* | 1.61E-04 | 2.21E-04 | 2.86E-04 | 3.92E-04 | 7.01E-04 | 1.15E-03 |
|  |  | PCB 101 | 3.20E-08 | 4.68E-08 | 6.17E-08 | 8.49E-08 | 1.53E-07 | 2.48E-07 |
|  |  | PCB 118 | 6.07E-08 | 7.38E-08 | 9.18E-08 | 1.24E-07 | 2.22E-07 | 3.81E-07 |
|  |  | PCB 128 | 5.57E-08 | 6.94E-08 | 8.68E-08 | 1.17E-07 | 2.08E-07 | 3.47E-07 |
|  |  | PCB 138 | 5.74E-08 | 6.95E-08 | 8.67E-08 | 1.17E-07 | 2.08E-07 | 3.57E-07 |
|  |  | PCB 153 | 1.15E-07 | 1.40E-07 | 1.74E-07 | 2.35E-07 | 4.24E-07 | 7.17E-07 |
|  |  | PCB 156 | 5.64E-08 | 7.24E-08 | 9.16E-08 | 1.23E-07 | 2.19E-07 | 3.53E-07 |
|  |  | PCB 163 | 5.09E-08 | 6.35E-08 | 7.93E-08 | 1.07E-07 | 1.91E-07 | 3.16E-07 |
|  |  | PCB 170 | 5.82E-08 | 7.17E-08 | 8.98E-08 | 1.21E-07 | 2.17E-07 | 3.59E-07 |
|  |  | PCB 180 | 5.97E-08 | 7.23E-08 | 9.04E-08 | 1.22E-07 | 2.18E-07 | 3.78E-07 |
|  |  | PCB 183 | 5.83E-08 | 7.19E-08 | 9.03E-08 | 1.21E-07 | 2.16E-07 | 3.68E-07 |
|  |  | PCB 187 | 6.00E-08 | 7.29E-08 | 9.12E-08 | 1.22E-07 | 2.21E-07 | 3.75E-07 |
|  |  | PCB 194 | 3.03E-08 | 6.23E-08 | 9.34E-08 | 1.35E-07 | 2.46E-07 | 3.95E-07 |
|  |  | PCB 28 | 5.68E-08 | 7.10E-08 | 8.90E-08 | 1.20E-07 | 2.12E-07 | 3.60E-07 |
|  |  | PCB 52 | 4.65E-08 | 5.82E-08 | 7.30E-08 | 9.83E-08 | 1.75E-07 | 3.02E-07 |
|  |  | PCB 99 | 4.61E-08 | 5.75E-08 | 7.18E-08 | 9.68E-08 | 1.73E-07 | 2.90E-07 |
|  |  | p-p’-DDD | 3.28E-09 | 5.53E-09 | 7.73E-09 | 1.09E-08 | 1.98E-08 | 3.23E-08 |
|  |  | p-p’-DDE | 1.04E-08 | 1.26E-08 | 1.58E-08 | 2.12E-08 | 3.83E-08 | 6.53E-08 |
|  |  | p-p’-DDT | 4.92E-09 | 7.45E-09 | 9.99E-09 | 1.39E-08 | 2.55E-08 | 3.96E-08 |
|  |  | β-Hexachlorocyclohexane | 1.05E-08 | 3.86E-08 | 7.56E-08 | 1.18E-07 | 2.22E-07 | 3.62E-07 |
|  |  | Toxaphene Parlar 26 | 1.01E-08 | 3.19E-08 | 5.37E-08 | 7.99E-08 | 1.47E-07 | 2.39E-07 |
|  |  | Toxaphene Parlar 32 | 7.26E-14 | 1.02E-09 | 5.28E-08 | 9.78E-08 | 1.82E-07 | 2.75E-07 |
|  |  | Toxaphene Parlar 50 | 1.05E-08 | 3.11E-08 | 5.28E-08 | 7.80E-08 | 1.46E-07 | 2.35E-07 |
|  |  | Trans-nonachlor | 5.11E-09 | 8.57E-09 | 1.20E-08 | 1.70E-08 | 3.05E-08 | 5.04E-08 |
|  |  |  |  |  |  |  |  |  |
|  | Women | Cis-nonachlor | 9.03E-09 | 1.45E-08 | 1.94E-08 | 2.53E-08 | 3.79E-08 | 6.66E-08 |
|  |  | Mirex* | 2.62E-04 | 3.27E-04 | 3.87E-04 | 4.63E-04 | 6.43E-04 | 1.19E-03 |
|  |  | Oxychlordane | 1.76E-08 | 2.28E-08 | 2.74E-08 | 3.33E-08 | 4.63E-08 | 8.84E-08 |
|  |  | PBB 153 | 2.36E-07 | 5.15E-07 | 7.66E-07 | 1.04E-06 | **1.57E-06** | 2.83E-06 |
|  |  | PBDE 100* | 4.74E-04 | 6.07E-04 | 7.24E-04 | 8.78E-04 | 1.23E-03 | 2.32E-03 |
|  |  | PBDE 153* | 5.43E-04 | 6.98E-04 | 8.36E-04 | 1.02E-03 | 1.42E-03 | 2.77E-03 |
|  |  | PBDE 154* | 2.96E-04 | 5.13E-04 | 7.04E-04 | 9.41E-04 | 1.40E-03 | 2.60E-03 |
|  |  | PBDE 47* | 3.18E-04 | 4.24E-04 | 5.19E-04 | 6.42E-04 | 9.21E-04 | 1.74E-03 |
|  |  | PCB 101 | 6.31E-08 | 8.89E-08 | 1.12E-07 | 1.40E-07 | 2.03E-07 | 3.64E-07 |
|  |  | PCB 118 | 1.23E-07 | 1.46E-07 | 1.67E-07 | 1.99E-07 | 2.70E-07 | 5.20E-07 |
|  |  | PCB 128 | 1.12E-07 | 1.36E-07 | 1.58E-07 | 1.88E-07 | 2.57E-07 | 4.88E-07 |
|  |  | PCB 138 | 1.15E-07 | 1.37E-07 | 1.57E-07 | 1.87E-07 | 2.52E-07 | 4.94E-07 |
|  |  | PCB 153 | 2.33E-07 | 2.76E-07 | 3.15E-07 | 3.76E-07 | 5.11E-07 | 9.91E-07 |
|  |  | PCB 156 | 1.15E-07 | 1.43E-07 | 1.66E-07 | 2.00E-07 | 2.77E-07 | 5.11E-07 |
|  |  | PCB 163 | 1.02E-07 | 1.24E-07 | 1.44E-07 | 1.73E-07 | 2.36E-07 | 4.58E-07 |
|  |  | PCB 170 | 1.18E-07 | 1.42E-07 | 1.63E-07 | 1.95E-07 | 2.63E-07 | 5.03E-07 |
|  |  | PCB 180 | 1.20E-07 | 1.43E-07 | 1.63E-07 | 1.95E-07 | 2.64E-07 | 5.18E-07 |
|  |  | PCB 183 | 1.18E-07 | 1.42E-07 | 1.64E-07 | 1.95E-07 | 2.67E-07 | 5.04E-07 |
|  |  | PCB 187 | 1.21E-07 | 1.44E-07 | 1.65E-07 | 1.96E-07 | 2.67E-07 | 5.21E-07 |
|  |  | PCB 194 | 5.89E-08 | 1.17E-07 | 1.69E-07 | 2.29E-07 | 3.43E-07 | 5.92E-07 |
|  |  | PCB 28 | 1.14E-07 | 1.39E-07 | 1.62E-07 | 1.93E-07 | 2.67E-07 | 4.93E-07 |
|  |  | PCB 52 | 9.34E-08 | 1.14E-07 | 1.33E-07 | 1.59E-07 | 2.16E-07 | 4.14E-07 |
|  |  | PCB 99 | 9.34E-08 | 1.13E-07 | 1.31E-07 | 1.56E-07 | 2.14E-07 | 4.04E-07 |
|  |  | p-p’-DDD | 6.38E-09 | 1.03E-08 | 1.38E-08 | 1.84E-08 | 2.77E-08 | 4.84E-08 |
|  |  | p-p’-DDE | 2.10E-08 | 2.50E-08 | 2.84E-08 | 3.38E-08 | 4.63E-08 | 9.03E-08 |
|  |  | p-p’-DDT | 9.74E-09 | 1.41E-08 | 1.80E-08 | 2.30E-08 | 3.38E-08 | 6.33E-08 |
|  |  | β-Hexachlorocyclohexane | 1.82E-08 | 7.06E-08 | 1.35E-07 | 2.07E-07 | 3.25E-07 | 5.40E-07 |
|  |  | Toxaphene Parlar 26 | 1.89E-08 | 5.79E-08 | 9.67E-08 | 1.39E-07 | 2.09E-07 | 3.60E-07 |
|  |  | Toxaphene Parlar 32 | 7.03E-14 | 2.05E-09 | 9.71E-08 | 1.77E-07 | 2.55E-07 | 4.67E-07 |
|  |  | Toxaphene Parlar 50 | 2.00E-08 | 5.81E-08 | 9.75E-08 | 1.39E-07 | 2.08E-07 | 3.73E-07 |
|  |  | Trans-nonachlor | 9.80E-09 | 1.61E-08 | 2.18E-08 | 2.86E-08 | 4.31E-08 | 7.67E-08 |
|  |  |  |  |  |  |  |  |  |
|  | Men | Cis-nonachlor | 1.31E-08 | 2.13E-08 | 2.84E-08 | 3.70E-08 | 5.27E-08 | 6.64E-08 |
|  |  | Mirex* | 3.89E-04 | 4.90E-04 | 5.78E-04 | 6.79E-04 | 8.78E-04 | 1.09E-03 |
|  |  | Oxychlordane | 2.61E-08 | 3.40E-08 | 4.05E-08 | 4.86E-08 | 6.32E-08 | 7.90E-08 |
|  |  | PBB 153 | 3.48E-07 | 7.63E-07 | 1.12E-06 | 1.53E-06 | **2.17E-06** | 2.75E-06 |
|  |  | PBDE 100* | 7.05E-04 | 9.06E-04 | 1.07E-03 | 1.28E-03 | 1.68E-03 | 2.09E-03 |
|  |  | PBDE 153* | 8.16E-04 | 1.04E-03 | 1.25E-03 | 1.48E-03 | 1.94E-03 | 2.40E-03 |
|  |  | PBDE 154* | 4.50E-04 | 7.71E-04 | 1.04E-03 | 1.37E-03 | 1.94E-03 | 2.49E-03 |
|  |  | PBDE 47* | 4.70E-04 | 6.36E-04 | 7.74E-04 | 9.42E-04 | 1.26E-03 | 1.57E-03 |
|  |  | PCB 101 | 9.27E-08 | 1.31E-07 | 1.65E-07 | 2.07E-07 | 2.80E-07 | 3.56E-07 |
|  |  | PCB 118 | 1.81E-07 | 2.19E-07 | 2.50E-07 | 2.87E-07 | 3.67E-07 | 4.84E-07 |
|  |  | PCB 128 | 1.67E-07 | 2.04E-07 | 2.37E-07 | 2.75E-07 | 3.50E-07 | 4.34E-07 |
|  |  | PCB 138 | 1.71E-07 | 2.06E-07 | 2.36E-07 | 2.69E-07 | 3.44E-07 | 4.52E-07 |
|  |  | PCB 153 | 3.45E-07 | 4.15E-07 | 4.76E-07 | 5.41E-07 | 6.91E-07 | 9.11E-07 |
|  |  | PCB 156 | 1.70E-07 | 2.12E-07 | 2.48E-07 | 2.90E-07 | 3.75E-07 | 4.70E-07 |
|  |  | PCB 163 | 1.52E-07 | 1.86E-07 | 2.16E-07 | 2.51E-07 | 3.22E-07 | 4.05E-07 |
|  |  | PCB 170 | 1.75E-07 | 2.12E-07 | 2.44E-07 | 2.82E-07 | 3.60E-07 | 4.60E-07 |
|  |  | PCB 180 | 1.79E-07 | 2.15E-07 | 2.46E-07 | 2.80E-07 | 3.58E-07 | 4.67E-07 |
|  |  | PCB 183 | 1.75E-07 | 2.13E-07 | 2.45E-07 | 2.83E-07 | 3.63E-07 | 4.57E-07 |
|  |  | PCB 187 | 1.78E-07 | 2.16E-07 | 2.49E-07 | 2.83E-07 | 3.63E-07 | 4.81E-07 |
|  |  | PCB 194 | 8.55E-08 | 1.73E-07 | 2.50E-07 | 3.34E-07 | 4.76E-07 | 5.93E-07 |
|  |  | PCB 28 | 1.71E-07 | 2.08E-07 | 2.43E-07 | 2.82E-07 | 3.58E-07 | 4.52E-07 |
|  |  | PCB 52 | 1.40E-07 | 1.71E-07 | 1.99E-07 | 2.32E-07 | 2.96E-07 | 3.78E-07 |
|  |  | PCB 99 | 1.38E-07 | 1.69E-07 | 1.96E-07 | 2.27E-07 | 2.91E-07 | 3.66E-07 |
|  |  | p-p’-DDD | 9.51E-09 | 1.53E-08 | 2.07E-08 | 2.69E-08 | 3.81E-08 | 4.84E-08 |
|  |  | p-p’-DDE | 3.11E-08 | 3.76E-08 | 4.30E-08 | 4.86E-08 | 6.21E-08 | 8.38E-08 |
|  |  | p-p’-DDT | 1.44E-08 | 2.11E-08 | 2.69E-08 | 3.39E-08 | 4.64E-08 | 5.91E-08 |
|  |  | β-Hexachlorocyclohexane | 2.80E-08 | 1.03E-07 | 2.00E-07 | 3.07E-07 | 4.57E-07 | 5.79E-07 |
|  |  | Toxaphene Parlar 26 | 2.76E-08 | 8.55E-08 | 1.44E-07 | 2.03E-07 | 2.92E-07 | 3.71E-07 |
|  |  | Toxaphene Parlar 32 | 1.88E-13 | 2.61E-09 | 1.42E-07 | 2.66E-07 | 3.61E-07 | 4.38E-07 |
|  |  | Toxaphene Parlar 50 | 2.79E-08 | 8.38E-08 | 1.41E-07 | 2.03E-07 | 2.87E-07 | 3.64E-07 |
|  |  | Trans-nonachlor | 1.49E-08 | 2.40E-08 | 3.20E-08 | 4.16E-08 | 5.88E-08 | 7.55E-08 |
|  |  |  |  |  |  |  |  |  |
| Goose | Girls | PCB 153 | 3.14E-09 | 4.36E-08 | 1.40E-07 | 2.81E-07 | 5.72E-07 | 1.01E-06 |
|  |  | p-p’-DDE | 1.41E-08 | 2.10E-08 | 2.73E-08 | 3.72E-08 | 6.50E-08 | 1.91E-07 |
|  |  |  |  |  |  |  |  |  |
|  | Boys | PCB 153 | 5.57E-09 | 6.56E-08 | 2.03E-07 | 3.89E-07 | 7.94E-07 | 1.36E-06 |
|  |  | p-p’-DDE | 2.14E-08 | 2.97E-08 | 3.86E-08 | 5.30E-08 | 9.47E-08 | 1.54E-07 |
|  |  |  |  |  |  |  |  |  |
|  | Women | PCB 153 | 6.79E-09 | 8.15E-08 | 2.50E-07 | 4.88E-07 | 8.20E-07 | 1.17E-06 |
|  |  | p-p’-DDE | 2.89E-08 | 3.90E-08 | 4.79E-08 | 5.93E-08 | 8.44E-08 | 1.59E-07 |
|  |  |  |  |  |  |  |  |  |
|  | Men | PCB 153 | 1.01E-08 | 1.23E-07 | 3.68E-07 | 7.18E-07 | **1.19E-06** | 1.52E-06 |
|  |  | p-p’-DDE | 4.37E-08 | 5.89E-08 | 7.20E-08 | 8.75E-08 | 1.17E-07 | 1.47E-07 |
|  |  |  |  |  |  |  |  |  |
| Walleye | Girls | Cis-nonachlor | 9.34E-17 | 6.07E-11 | 6.75E-09 | 1.17E-08 | 2.30E-08 | 3.55E-08 |
|  |  | PBDE 100* | 1.32E-10 | 2.23E-05 | 2.56E-04 | 3.62E-04 | 6.66E-04 | 2.17E-03 |
|  |  | PBDE 47* | 2.21E-07 | 3.04E-05 | 1.62E-04 | 2.99E-04 | 5.75E-04 | 1.01E-03 |
|  |  | PCB 118 | 1.91E-08 | 3.25E-08 | 4.44E-08 | 6.11E-08 | 1.09E-07 | 3.12E-07 |
|  |  | PCB 128 | 2.57E-09 | 4.22E-09 | 7.69E-09 | 1.31E-08 | 3.36E-08 | 5.65E-08 |
|  |  | PCB 138 | 3.81E-19 | 6.39E-10 | 5.22E-08 | 7.38E-08 | 1.34E-07 | 3.30E-07 |
|  |  | PCB 153 | 7.33E-11 | 1.43E-08 | 4.92E-08 | 7.02E-08 | 1.32E-07 | 2.97E-07 |
|  |  | PCB 170 | 1.42E-16 | 3.22E-09 | 5.32E-08 | 7.38E-08 | 1.39E-07 | 4.34E-07 |
|  |  | PCB 180 | 2.73E-16 | 2.82E-09 | 5.32E-08 | 7.41E-08 | 1.39E-07 | 4.34E-07 |
|  |  | PCB 187 | 5.79E-11 | 1.58E-08 | 5.14E-08 | 7.17E-08 | 1.32E-07 | 3.65E-07 |
|  |  | p-p’-DDE | 1.65E-11 | 1.63E-08 | 5.22E-08 | 7.33E-08 | 1.34E-07 | 4.24E-07 |
|  |  |  |  |  |  |  |  |  |
|  | Boys | Cis-nonachlor | 5.89E-16 | 2.95E-10 | 3.18E-08 | 5.16E-08 | 1.01E-07 | 1.41E-07 |
|  |  | PBDE 100* | 2.14E-09 | 9.92E-05 | 1.13E-03 | 1.64E-03 | 3.12E-03 | 4.37E-03 |
|  |  | PBDE 47* | 1.24E-06 | 1.28E-04 | 7.43E-04 | 1.32E-03 | 2.48E-03 | 3.97E-03 |
|  |  | PCB 118 | 9.04E-08 | 1.50E-07 | 2.01E-07 | 2.77E-07 | 5.06E-07 | 7.46E-07 |
|  |  | PCB 128 | 1.16E-08 | 2.14E-08 | 3.77E-08 | 6.61E-08 | 1.17E-07 | 1.83E-07 |
|  |  | PCB 138 | 1.24E-17 | 3.24E-09 | 2.37E-07 | 3.33E-07 | 6.27E-07 | 8.56E-07 |
|  |  | PCB 153 | 4.11E-10 | 6.37E-08 | 2.19E-07 | 3.17E-07 | 5.93E-07 | 8.50E-07 |
|  |  | PCB 170 | 2.13E-15 | 1.63E-08 | 2.46E-07 | 3.41E-07 | 6.37E-07 | 8.81E-07 |
|  |  | PCB 180 | 1.61E-15 | 1.45E-08 | 2.44E-07 | 3.36E-07 | 6.27E-07 | 8.73E-07 |
|  |  | PCB 187 | 3.88E-10 | 8.13E-08 | 2.31E-07 | 3.29E-07 | 6.11E-07 | 8.66E-07 |
|  |  | p-p’-DDE | 1.11E-10 | 7.82E-08 | 2.35E-07 | 3.30E-07 | 6.27E-07 | 8.81E-07 |
|  |  |  |  |  |  |  |  |  |
|  | Women | Cis-nonachlor | 1.54E-15 | 4.27E-10 | 4.79E-08 | 7.43E-08 | 1.05E-07 | 1.94E-07 |
|  |  | PBDE 100* | 1.80E-09 | 1.62E-04 | 1.80E-03 | 2.27E-03 | 3.12E-03 | 5.97E-03 |
|  |  | PBDE 47* | 1.99E-06 | 1.90E-04 | 1.08E-03 | 1.91E-03 | 2.81E-03 | 5.01E-03 |
|  |  | PCB 118 | 1.39E-07 | 2.24E-07 | 2.92E-07 | 3.65E-07 | 5.15E-07 | 9.74E-07 |
|  |  | PCB 128 | 1.98E-08 | 2.87E-08 | 4.75E-08 | 8.22E-08 | 1.50E-07 | 2.51E-07 |
|  |  | PCB 138 | 1.29E-17 | 5.09E-09 | 3.75E-07 | 4.62E-07 | 6.26E-07 | 1.11E-06 |
|  |  | PCB 153 | 6.88E-10 | 9.55E-08 | 3.41E-07 | 4.41E-07 | 6.11E-07 | 1.16E-06 |
|  |  | PCB 170 | 2.84E-15 | 2.40E-08 | 3.78E-07 | 4.65E-07 | 6.42E-07 | 1.22E-06 |
|  |  | PCB 180 | 5.80E-15 | 2.15E-08 | 3.77E-07 | 4.65E-07 | 6.40E-07 | 1.22E-06 |
|  |  | PCB 187 | 4.99E-10 | 1.06E-07 | 3.57E-07 | 4.51E-07 | 6.08E-07 | 1.11E-06 |
|  |  | p-p’-DDE | 1.64E-10 | 1.12E-07 | 3.68E-07 | 4.57E-07 | 6.40E-07 | 1.19E-06 |
|  |  |  |  |  |  |  |  |  |
|  | Men | Cis-nonachlor | 1.18E-15 | 5.89E-10 | 6.97E-08 | 1.09E-07 | 1.46E-07 | 1.81E-07 |
|  |  | PBDE 100* | 4.45E-09 | 1.98E-04 | 2.65E-03 | 3.37E-03 | 4.34E-03 | 5.22E-03 |
|  |  | PBDE 47* | 2.48E-06 | 2.52E-04 | 1.54E-03 | 2.79E-03 | 3.83E-03 | 4.86E-03 |
|  |  | PCB 118 | 2.00E-07 | 3.29E-07 | 4.25E-07 | 5.28E-07 | 7.04E-07 | 8.78E-07 |
|  |  | PCB 128 | 2.67E-08 | 3.90E-08 | 7.00E-08 | 1.23E-07 | 2.02E-07 | 2.47E-07 |
|  |  | PCB 138 | 2.16E-17 | 6.69E-09 | 5.50E-07 | 6.85E-07 | 8.89E-07 | 1.04E-06 |
|  |  | PCB 153 | 9.10E-10 | 1.29E-07 | 4.87E-07 | 6.45E-07 | 8.43E-07 | 1.03E-06 |
|  |  | PCB 170 | 4.15E-15 | 3.33E-08 | 5.58E-07 | 6.89E-07 | 8.94E-07 | 1.05E-06 |
|  |  | PCB 180 | 3.33E-15 | 2.81E-08 | 5.57E-07 | 6.89E-07 | 8.94E-07 | 1.05E-06 |
|  |  | PCB 187 | 6.93E-10 | 1.64E-07 | 5.26E-07 | 6.69E-07 | 8.68E-07 | 1.04E-06 |
|  |  | p-p’-DDE | 2.35E-10 | 1.60E-07 | 5.40E-07 | 6.73E-07 | 8.63E-07 | 1.04E-06 |

*Key*:

* indicates hazard not risk as the contaminant has no slope factor

P*_n_*: *n^t^*^h^-percentile

Bold indicates P_95_ risk at or above 1.00 x 10^-6^
