# Supplementary material for: A probabilistic hazard and risk assessment of exposure to metals and organohalogens associated with a traditional diet in the Indigenous communities of Eeyou Istchee (northern Quebec, Canada)
Source: Environ Sci Pollut Res Int. 2022 Sep 24;30(6):14304–17. doi: 10.1007/s11356-022-23117-2 (PMC9908690; doi:10.1007/s11356-022-23117-2)
Supplement: Supplementary file 7 — (DOCX 14 kb) [file 11356_2022_23117_MOESM7_ESM.docx]

**Table S7: Sensitivity analysis results (Spearman’s rho) for organohalogens in traditional foods with R_95_ greater than 1.00 x 10^-6^ by consumer group.**

|  | Traditional Food | | | |
| --- | --- | --- | --- | --- |
|  | Goose | Duck | | |
| Organohalogen: | PCB 153 | PBB 153 | | |
|  | Men | Boys | Women | Men |
| IR | **0.53** | **0.39** | **0.44** | **0.38** |
| *C* | **0.78** | **0.88** | **0.89** | **0.90** |
| EF | 0.02 | -0.03 | -0.02 | -0.01 |
| ED | 0.01 | 0.01 | -0.01 | -0.11 |
| BW | **-0.13** | **-0.19** | **-0.12** | **-0.12** |
| AT | -0.01 | 0.01 | -0.01 | 0.02 |

*Key*: IR (intake rate); *C* (contaminant concentration); EF (exposure frequency); ED (exposure duration); BW (body weight); AT (averaging time).

*Note*: bold indicates result is statistically significant at p < 0.05.
